# Mosaic patterns of diversification dynamics following the colonization of Melanesian islands

**DOI:** 10.1038/srep16016

**Published:** 2015-11-03

**Authors:** Emmanuel F. A. Toussaint, Lars Hendrich, Helena Shaverdo, Michael Balke

**Affiliations:** 1 SNSB-Bavarian State Collection of Zoology, Münchhausenstraβe 21, 81247 Munich, Germany; 2 Department of Ecology & Evolutionary Biology & Division of Entomology, Biodiversity Institute, University of Kansas, Lawrence, KS 66045, USA; 3 Naturhistorisches Museum, Burgring 7, A-1010 Vienna, Austria; 4 GeoBioCenter, Ludwig-Maximilians University, Munich, Germany

## Abstract

The fate of newly settled dispersers on freshly colonized oceanic islands is a central theme of island biogeography. The emergence of increasingly sophisticated methods of macroevolutionary pattern inference paves the way for a deeper understanding of the mechanisms governing these diversification patterns on lineages following their colonization of oceanic islands. Here we infer a comprehensive molecular phylogeny for Melanesian *Exocelina* diving beetles. Recent methods in historical biogeography and diversification rate inference were then used to investigate the evolution of these insects in space and time. An Australian origin in the mid-Miocene was followed by independent colonization events towards New Guinea and New Caledonia in the late Miocene. One colonization of New Guinea led to a large radiation of >150 species and 3 independent colonizations of New Caledonia gave rise to about 40 species. The comparably late colonizations of Vanuatu, Hawaii and China left only one or two species in each region. The contrasting diversification trajectories of these insects on Melanesian islands are likely accounted for by island size, age and availability of ecological opportunities during the colonization stage.

According to the dynamic equilibrium theory of island biogeography, species richness and turnover are functions of immigration and extinction on one hand and area size and isolation on the other^1^,^2^. However, in order to provide a simple and widely-relevant model, MacArthur and Wilson^1^,^2^ ignored island history. Many have called for this decision to be readdressed in order to better predict patterns of diversity across oceanic islands^3^,^4^,^5^. Recent studies have paved the way to better understand population dynamics within the framework of this theory^5^,^6^,^7^,^8^. However, comparative studies investigating the diversification dynamics of oceanic island colonizers at a macroevolutionary scale remain sparse. In the past decade, new approaches were developed to infer macroevolutionary patterns in a phylogenetic framework. These methods allow for testing which factors shape biodiversity assembly in space and time using an array of birth-death models of diversification^9^,^10^ and more explicit biogeographic models^11^,^12^,^13^,^14^,^15^.

In oceanic settings, speciation (anagenetic and cladogenetic) and geological history of an island are crucial to understand diversification mechanisms^5^,^8^,^16^. Theoretically, remote islands should house larger amounts of endemics and exhibit higher rates of *in situ* diversification as immigration rates decline with isolation. As a result, vacant ecological niches (i.e. overlap of an ecological opportunity and species properties) are progressively filled through adaptive diversification of the rare successful immigrants. In contrast, islands closer to continental landmasses are expected to have fewer endemics because the ratio of immigration to *in situ* diversification is higher (“radiation zone” hypothesis^2^). Additionally, recent colonizers are expected to be less diversified *in situ* than older taxa occurring on the same island^17^. Hence, the ontogeny of an oceanic island has a major impact on the evolutionary trajectory of its biota because the cladogenetic speciation rate is supposed to be negatively correlated with the age of the island^5^.

Situated in the inter-tropical belt, the Indomalayan-Australasian archipelago (IAA) has a highly complex geological history. Most of the present-day island arrangements are the result of plate collisions and extensive volcanism throughout the Cenozoic^18^,^19^. The Melanesian archipelago, which extends the IAA towards the east, has particularly fascinated evolutionary biologists because of the unique geological past and extraordinary biological diversity of its islands.

High rates of endemism and the presence of a few enigmatic species on New Caledonia have been explained either by persistence of ancient Gondwanean lineages (Gondwanean refugium hypothesis^20^,^21^) or recent colonization events (Darwinian island hypothesis^22^,^23^). New Caledonia separated from Gondwana *c*. 65 million years ago (Ma), and was possibly completely submerged until 37M^24^. Australia on the other hand remained part of Gondwana along with parts of New Guinea, India and Antarctica until it began to drift northwards *c*. 55 Ma^18^. This island has never been submerged and until the onset of a severe Miocenic aridification^25^, geology and climate remained steady. New Guinea has a highly dynamic geological history including a complex late-Miocenic orogeny, oceanic island arc accretion, volcanism and docking of Australian continental fragments^26^. These different properties enable the three islands with their unique biota to serve as a laboratory for comparative biogeography and empirical studies of diversification processes.

Here we focus on *Exocelina* diving beetles (Coleoptera, Dytiscidae, Copelatinae), which have their 141 mostly endemic species concentrated in New Guinea, New Caledonia and Australia, with one or two species in each China, Hawaii and Vanuatu. Species diversity is also unevenly distributed between Australia (15 described species and *ca*. 15 undescribed ones), New Caledonia (37 described species and *ca.* 10 undescribed ones) and New Guinea (88 described species and more than 70 undescribed ones). Most species are associated with riparian habitats such as puddles at the edge of streams, though a few species occupy stagnant-water habitats and even Australian underground aquifers. While aspects of their historical biogeography and diversification dynamics have been studied recently, a comprehensive synthesis had yet to be undertaken^26^,^27^,^28^.

In this study, we assembled an eight-marker dataset for 164 *Exocelina* species (≈70% of the described diversity plus several undescribed species). We aim to: (*i*) reconstruct a robust phylogenetic hypothesis for the group; (*ii*) use fossil-calibrated divergence time estimate analyses to understand their evolution in time; (*iii*) present a historical biogeography hypothesis to reveal their evolution in space from a macroevolutionary perspective; and (*iv*) test if their post-colonization diversification dynamics are similar on different islands with very different geological histories.

In particular, we aim to test the following hypotheses derived from island biogeography predictions:

1. The Australian biota is comparably ancient and the source of colonization events to surrounding islands. Due to the mid-Miocene aridification in Australia, diversification rates might have decreased as shown in a different group of diving beetles^29^.
2. Colonization of New Caledonia was rare and diversification rates should have decreased as available habitats were mainly being filled by cladogenetic speciation.
3. The New Guinean biota comes from multiple colonizations events and diversification rates should be constant or increasing as the island is continuously increasing in area, altitude and topographic complexity since the mid-Miocene.

## Results

**Figure 1:**
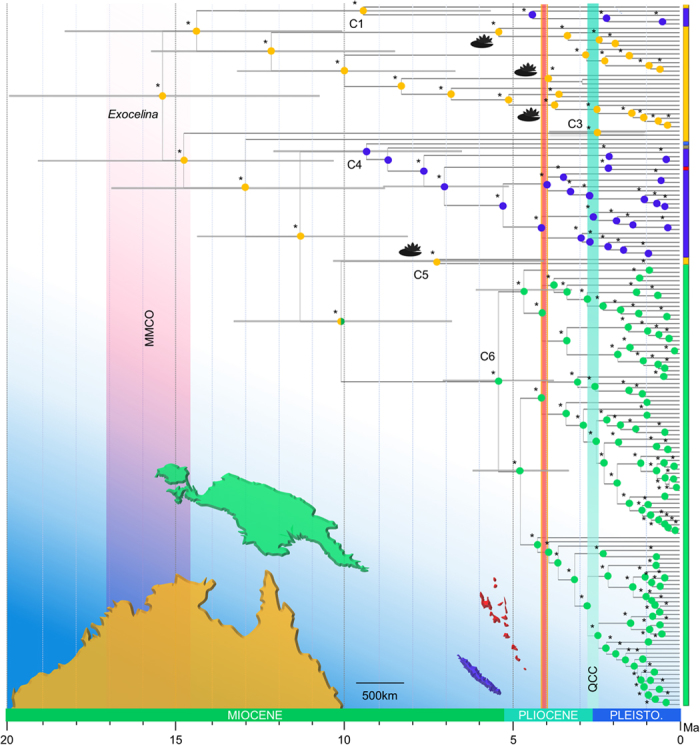
Historical biogeography of *Exocelina* predaceous diving beetles. BEAST chronogram presenting the median ages with 95% height posterior distribution of major nodes. The bottom of the figure represents at a geographic scale the four Oceanian territories where the genus *Exocelina* is distributed; Australia (yellow), New Guinea (green), New Caledonia (violet) and Vanuatu (red). At the tips of the chronogram, vertical bars matching the colors of these regions indicate the present-day distribution of species according to the delineation used in the BioGeoBEARS analyses. China and Hawaii which are not represented on this map and are respectively coded in brown and blue. Colored pastilles at each node correspond to the highest probability range as recovered by the best BioGeoBEARS model selected with Bayes factors (DIVALIKE+J model). Two vertical bars represent the following major climatic events: Mid-Miocene Climatic Optimum (MMCO) and QCC (Quaternary Climatic Change). Asterisks indicate a relative probability >80% for the ancestral range. Clades comprising exclusively lentic species are annotated with a water lily symbol. All other clades comprise riparian species. The geographic map was drawn in Microsoft PowerPoint based on a blank map of the World freely available on the website https://commons.wikimedia.org/.

Altogether, the Maximum Likelihood (ML) and Bayesian Inference (BI) topologies respectively resulting from the RAxML and MrBayes analyses were congruent. We recover all Copelatinae genera as monophyletic with strong support in both BI and ML (Table 1; Supplementary Figures S1 and S2). In BI *Capelatus* is recovered as sister to *Exocelina* whereas in ML it forms a sister clade to *Exocelina *+ *Liopterus*. Supports in BI and ML are moderate or low for the placement of these taxa between each other although *Exocelina* is always found well delineated from the rest of the genera. Within *Exocelina*, most of the nodes are very well supported except for two early nodes due to the changing placement of two small clades labeled C1 and C3 (Table 1; Fig. 1; Supplementary Figures S1 and S2). Australian and New Caledonian *Exocelina* are not monophyletic. Clades C2 and C4 contain most of the diversity of Australia and New Caledonia. *Exocelina parvula* from Hawaii and *E. shizong* from China are found as sister to the largest clade of New Caledonian species in C4 whereas *E. cheesmanae* from Vanuatu is found nested in this clade. The morphologically rather deviant *E. abdita* (stygobiont) and *E. elongatula* are in clade C5 as sister to all New Guinean *Exocelina* which are recovered in a monophyletic clade C6 with strong support (Supplementary Figures S1 and S2).

We find a Cretaceous origin for Copelatinae *c*. 73.50 Ma (95HPD: 57.02 – 95.21 Ma) and a mid-Miocene origin of *Exocelina c*. 15.35 Ma (95%HPD: 11.20 – 19.92 Ma) (Fig. 1). The best biogeographical model recovered under the program BioGeoBEARS (Table 2) is the DEC+J model (AIC = 93.94, −logL = 43.97) implementing a founder-effect component presented in Fig. 1, although it is not significantly better than the DIVALIKE+J model (AIC = 93.97, −logL = 43.98). The only difference in the biogeographical pattern recovered is the geographic range at the ancestor of C5 and C6, which is Australia in DIVALIKE+J and Australia + New Guinea in DEC+J. Overall, all models implementing a founder-effect parameter were significantly better than the same models without this component (Table 2). Our results suggest an Australian origin of *Exocelina* followed by two independent colonizations of New Caledonia during the late Miocene respectively in C1 and C4 roughly between 5 and 10 Ma. The colonization of New Guinea out of Australia are suggested to have occurred at the same time. The colonizations of China, Hawaii and Vanuatu were suggested to be recent more or less long-distance dispersal events out of New Caledonia in C4.

**Figure 2:**
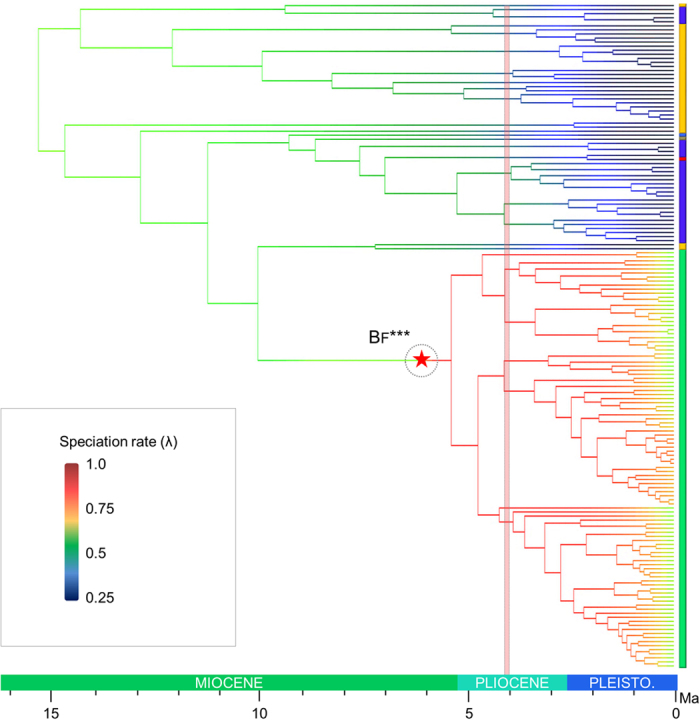
Diversification dynamics of *Exocelina* predaceous diving beetles. Phylorate extrapolated from the BAMM analyses where branch colors represent speciation rates following the scale inserted on the left of the figure. The speciation rate shift recovered in BAMM analyses is indicated by a red star in a green circle. A red vertical bar indicates the diversification rate shift recovered in the *TreePar* analyses. The Phylorate was drawn in R.

The best model of diversification rate dynamics recovered in the *TreePar* analysis is a model with a unique diversification shift (−logL = 199.856, *p*-value = 0.008 compared to the constant rate model) in the mid-Pliocene around 4.1 Ma (Fig. 2, Supplementary Table S1). This model is significantly better than a constant-rate model or other diversification models with varying rates. Using a diversity of 300 species instead of the 198 known to us, we obtain similar results with a significantly better model including a unique shift around 4.1 Ma. Under this model *Exocelina* would have had an initially slow diversification rate between 15.35 Ma and 4.1 Ma before entering a stage of high diversification that is still ongoing. The model recovers a slight turnover in the early evolution of the group and no turnover after the diversification rate shift.

**Figure 3:**
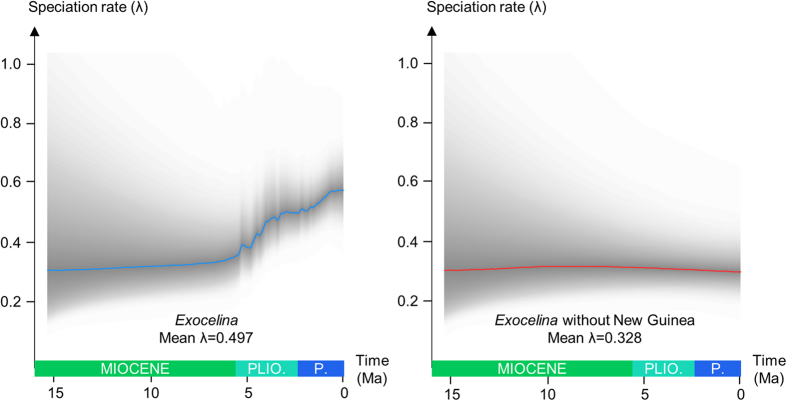
Speciation rate dynamics of the *Exocelina* radiation. Evolution of speciation rates in different subsets of the *Exocelina* phylogeny based on the results of the BAMM analyses. Plain lines indicate the mean speciation rate throughout the evolution of the clade. Shaded areas indicate the 95% credibility interval of the calculated speciation rate.

**Figure 4:**
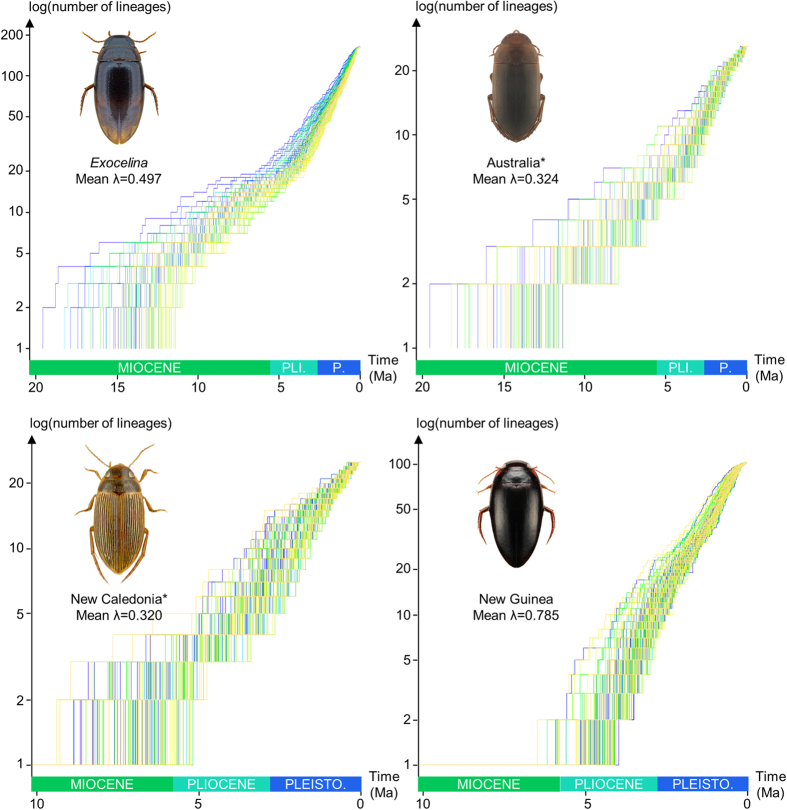
Clade-specific lineage-through-time plots across the *Exocelina* radiation. Australia* refers to clade C2, New Caledonia* refers to clade C5. Habitus pictures of the following species: *E. parvula* from Hawaii, *E. australis* from Australia, *E. perfecta* from New Caledonia and an undescribed species from New Guinea. The habitus pictures were taken by the authors with collection specimens stored in the Bavarian State Collection of Zoology in Munich. The LTT plots were drawn in R.

The diversification rate analyses conducted in the program BAMM (Bayesian Analysis of Macroevolutionary Mixtures) also recovered one significantly supported shift localized in C4 at the root of the New Guinean radiation (Fig. 2). Our clade-specific analyses are presented in Figs 3 and 4. Although the reduced Australian and New Caledonian clades are found with a similar speciation rate (respectively λ = 0.324 and λ = 0.320), the New Guinean radiation has a much faster speciation rate (λ = 0.785) whereas the global *Exocelina* radiation has an intermediate value (λ = 0.497) (Fig. 3). As highlighted in Figs 3 and 4, the New Guinean radiation is responsible for a large part of the speciation rate under the BAMM model with a calculated speciation rate for *Exocelina* of λ = 0.328 without the New Guinean radiation.

The best model recovered in the diversification rate analyses conducted in MuSSE (Multiple State Speciation Extinction) regarding the diversification pattern of the islands suggests different speciation rates but equal extinction and transition states (AIC = 648.46, −logL = 318.23, Supplementary Table S2). As a result, the best model supports the hypothesis of different speciation rates depending on the island the beetles are or have colonized. The second best model recovered is similar to the best model but it also relaxes the extinction parameter so that the different islands have both different speciation and extinction rates (AIC = 653.16, −logL = 317.58). One model (λ1, μ1 and q1 free) consistently crashed in the program R and as result was not included in the global comparison of models although considering the likelihood and AIC scores of the closest models it seems unlikely that this model might have been the best.

## Discussion

We confirm an Australian origin for the genus *Exocelina*^28^ and refine the temporal sequence during which these beetles colonized surrounding regions and diversified. The ancestor of the *Exocelina* radiation originated in Australia in the mid-Miocene. Our results are in agreement with previous studies using different calibration priors to obtain absolute divergence ages. Balke *et al.*^28^ found an origin of *Exocelina* at 23.34  Ma (95% HPD: 20.8–25.9 Ma) using a calibration on the node connecting the stygobiont species *E. abdita* and its sister lineage accounting for the timing of underground aquifer invasion in Australia. More recently Toussaint *et al.*^26^ calculated an age of 15.4 Ma (95% HPD: 10.3–22.1 Ma) using a combination of mitochondrial substitution rates calculated for adephagan beetles. These estimates are broadly overlapping and strongly support a mid-Miocene origin of the genus. Here we suggest that founder event speciation processes defined here as cladogenesis following the colonization of a new island have been important in the biogeographical history of *Exocelina* as expected in an archipelagic setting^14^. The best model suggests colonization of New Caledonia and New Guinea in the late Miocene within roughly 10 Myr. Additionally, recent taxonomic work revealed one lentic species (*E. baliem*) in New Guinea which we were unable to sequence but places together with the Australian *E. ferruginea* in C2 based on morphology^30^. Likewise, the New Caledonian fauna comprises the lentic *E. inexpectata* known from the holotype only and that is closely related to two above species based on an apomorphy from the male genital (paramere shape) therefore suggesting an additional colonization event of New Caledonia^30^. Both colonizations of lentic species would have occurred *c*. around 5 Ma.

The late Miocene emergence of New Guinea in addition to the enhanced connectivity between Australia and New Guinea in the Plio-Pleistocene^31^ might indicate an ecological opportunity scenario. The amount of emerging new habitats was very high during the New Guinea orogeny since 10 Ma^26^. The absence of reverse colonizations back to Australia^28^ might hint towards an absence of opportunity/open niches in northern Australia.

For New Caledonia, a scenario of long-distance dispersal caused by climatic disruptions might be more suitable with respect to the distance separating the island from Australia. Indeed, the only land bridge hypothesized between Australia and New Caledonia has been suggested to have existed until the Paleocene/Eocene^32^. This largely predates the colonization events out-of-Australia in our case. Long-distance dispersal events have rarely been documented in diving beetles but the distribution of some endemic clades and species in remote oceanic archipelagoes (e.g. Hawaii, Tristan da Cunha) seem to indicate that these beetles are good dispersers. Understanding if these events are the result of active (direct flight) or passive dispersal (e.g. as adults by rafting on tree logs or wind dispersal/as larvae in soil in which they pupate/as eggs in aquatic plant stems and leaves) would certainly bring new insights into our understanding of biogeographical and diversification processes in dytiscids.

All diversification rate analyses indicate a single shift in the evolution of the genus (Fig. 2). The BAMM analyses indicate that this shift took place when *Exocelina* colonized New Guinea in the late Miocene. This event comes comparably late in the evolution of *Exocelina*, as New Caledonia had already been colonized twice despite its more remote location.

Our results reject some of our initial hypotheses as well as predictions from the theory of island biogeography and reveal some interesting patterns for the region:

The two distinct New Caledonian clades do not exhibit particularly fast diversification rates compared to the New Guinean radiation. Interestingly, the diving beetle fauna of New Caledonia is rather diverse, with *Exocelina* accounting for more than 50% of its species, but there are no ecologically similar species^33^. This might indicate either that these beetles were able to diversify into vacant niches at the time of their arrival on the island, or that they were extremely efficient competitors that eliminated pre-existing indigenous taxa occupying the same niche. *Copelatus* are phylogenetically and ecologically close but only one stagnant water species occurs in New Caledonia^33^. Other genera are either not associated with running-water or ecologically well differentiated even in terms of body size and shape. Therefore, they were unlikely to represent competitors for niche filling. This is supported by the fact that *Exocelina* diving beetles are absent from Fiji where *Copelatus* diving beetles are quite diverse occupying the same type of habitat as *Exocelina*^34^. In addition to vacant niches, the association of these beetles with running-water habitats might have fuelled their diversification through local allopatric speciation^35^,^36^. Micro-endemism is likely a dominant process accounting for the diversification of *Exocelina*^26^. The ancestor of the second and larger running-water clade C4 might have brought new ecological preferences, helping them to compete and diversify on the island.

One colonization of New Guinea gave rise to a radiation of certainly more than 150 riparian species. This diversification has been very fast (Figs 1, 2, 3, 4) and serves as a prime example of *in situ* speciation as a source of (island) diversity^8^. This result is in contradiction with theoretical predictions as most of the diversity results from a unique colonization event followed by cladogenetisis rather than from multiple colonization events. As suggested by Toussaint *et al.*^26^, there is an intimate relationship between the massive orogeny of New Guinea and the diversification processes responsible for this diversification. The association of micro-endemism with high elevational gradients along the central Cordillera might have supported ecological (perhaps montane) speciation^37^,^38^ along elevational ecological gradients and/or allopatry in a complex vertical and horizontal setting. New Guinea *Exocelina* occur from almost sea level up to at least 2800 m^26^. Especially towards lower altitudes, they compete with ecologically very similar but slightly larger New Guinea *Copelatus* species (Balke pers. com.), which might explain the lack of larger New Guinea *Exocelina* species.

Our results reveal heterogeneous post-colonization diversification patterns on different Melanesian islands. Colonizers from the Australian source faced different levels of competition, niche preemption and habitat availability with these different parameters being shaped by the age and distance from the source of the island being colonized. Here we show that on the very recent and close landmass of New Guinea, a unique colonization event resulted in one of the most extensive beetle radiations of the region. The recent nature of that colonization and fast ongoing diversification are reflected by rather homogeneous morphology and lineage accumulation below saturation. Roughly 3,000 kilometers southeast, New Caledonia exhibits a rather different evolutionary history. Colonizers have reached the island on multiple occasions but unlike New Guinea, the island at the time of colonization hosted a variety of habitats and a diverse biota derived either from old Gondawanan stocks and/or from relatively old colonizers that settled in New Caledonia after its debated submergence^20^,^21^,^22^,^23^,^24^. If that was the case, the new colonizers were able to outcompete such fauna, and diversify extensively. Competition with a second *Exocelina* colonization might have fostered the extinction of the species of the older clade and pushed survivors to more extreme high altitude habitats. Our results broadly embrace the predictions of the theory of island biogeography but also bring new insights to our understanding of Melanesian biogeography and macroevolutionary patterns and processes. The study of well-established systems such as *Exocelina* should be moved to the next level where the use of comprehensive local sampling across environmental gradients and population genomics will help to reveal the actual ecological and evolutionary mechanisms of speciation on these islands^8^.

## Material and Methods

### Taxon sampling and molecular biology

We supplemented the datasets of Balke *et al.*^27^,^28^, Toussaint *et al.*^26^ and Bilton *et al.*^39^ with additional specimens and gene coverage and assembled a comprehensive dataset of 96 described and 68 undescribed *Exocelina* species (Supplementary Table S3). Total genomic DNA was extracted from whole beetles kept in 96% ethanol using a DNeasy kit (Qiagen, Hilden, Germany). We used PCR protocols following Toussaint *et al.*^26^ to amplify and sequence the following gene fragments: 18S rRNA (546 bp), alpha-spectrin (α-spec, 792 bp) carbamoyl phosphate synthetase 2 (CAD, 849 bp), cytochrome oxidase subunit 1 (COI, 732 bp), cytochrome oxidase subunit 2 (CO2, 552 bp), cytochrome b (Cytb, 306 bp), histone 3 (H3, 315 bp) and histone 4 (H4, 156 bp). The DNA sequences were edited in Geneious R6 (Biomatters, http://www.geneious.com/), aligned using Muscle^40^ and the reading frames checked in Mesquite 3.01 (http://mesquiteproject.org). The different datasets used to infer phylogenetic relationships were generated in Mesquite. New sequences were deposited in GenBank (accession Nos. HG973548-HG974146). The sequence matrix is also deposited at Dryad (doi:10.5061/dryad.d88dg).

### Molecular phylogenetics

We used Bayesian Inference (BI) and Maximum Likelihood (ML) to reconstruct phylogenetic relationships using a concatenated dataset. The partitions and corresponding optimal models of substitution were searched under PartitionFinder 1.1.1^41^ using the ‘*greedy’* algorithm. Both ‘*mrbayes’* and ‘*raxml’* sets of models were used in combination with the Akaike Information Criterion corrected (AICc) to compare the fit of the different models of substitution. All protein-coding gene fragments were divided by codon positions and non-coding gene fragments were kept alone. Since it is not currently possible to specify different models of substitution for different partitions in RAxML, we used the partitions selected in PartitionFinder with a GTR+Γ+I model. The BI analyses were performed using MrBayes3.2.2^42^. Instead of selecting the substitution models a priori based on the results of PartitionFinder, we used the different partitions recovered but used reversible-jump Metropolis-coupled Markov chain Monte Carlo to explore the entire space of substitution models^43^. Two simultaneous and independent runs consisting of eight MCMC (one cold and seven incrementally heated) running 100 million generations were used, with a tree sampling every 5000 generations to calculate posterior probabilities (PP). We assessed convergence of the runs by investigating the average standard deviation of split frequencies and Effective Sample Size (ESS) of all parameters in Tracer1.5 (http://BEAST.bio.ed.ac.uk/Tracer). A value of ESS > 200 was acknowledged as a good indicator of convergence. All posterior trees that predated the time needed to reach a log-likelihood plateau were discarded as burn-in, and the remaining samples were summarized to generate a 50% majority rule consensus tree. The ML analyses were conducted with the best partitioning scheme selected in PartitionFinder 1.1.1^41^ using RaxML^44^. We performed 1000 *Bootstrap* replicates (BS) to investigate the level of support at each node‘.

### Divergence time estimation

Divergence times were inferred with BEAST 1.8.0^45^. The partitions and models of nucleotide substitution were selected under PartitionFinder 1.1.1^41^ using the ‘*greedy’* algorithm, the ‘*beast’* set of models and the AICc. We tested the applicability of a molecular clock for both datasets using Paup*^46^, and since it was significantly rejected (*P *< 0.001), we used a Bayesian relaxed clock allowing rate variation among lineages as implemented in BEAST. In order to calibrate the tree, we used an amber fossil of the genus *Copelatus*. *Copelatus aphrodite*^†^ is an extinct species described based on a very-well preserved female specimen embedded in Baltic amber^47^. Because of its exceptional condition, the authors were able to place this species with confidence in the genus *Copelatus* even though it was not assigned to an extant species-group^47^. Because we sampled a substantial number of *Copelatus* species-group to capture a part of the variability in this diverse genus, we placed the fossil at the crown of *Copelatus*. Evidences from stratigraphic^48^ and K-Ar radiometric studies^49^ correlate and indicate that Baltic amber is of middle Lutetian Age, about 44.0 Ma^50^. We also placed a maximum constraint of 154.8 million years (Myr) at the root, an age equivalent to the oldest Dytiscid fossil known^51^. As a result, a minimum age of 44.0 Myr was placed at the crown of *Copelatus* with a soft exponential distribution prior (95% of the distribution between 44.0 and 154.8) as advocated for fossil information^52^. The runs performed under a *Birth*-*Death process* consisted of 100 million generations sampled every 2000 generations. The convergence of the runs was investigated using ESS, a conservative burn-in of 25% applied after checking the log-likelihood curves and the different runs merged using LogCombiner 1.8.0^45^. The maximum credibility tree, median ages and their 95% highest posterior density (HPD) were generated afterwards under TreeAnnotator 1.8.0^45^.

### Ancestral range estimation

We used BioGeoBEARS^53^ as implemented in R to infer the biogeographical history of *Exocelina* diving beetles across their entire range of distribution. This program allows estimation of ancestral ranges under different models such as DEC^11^,^12^, a likelihood interpretation of DIVA^54^ (DIVALIKE) or a likelihood interpretation of BayArea^13^ (BAYAREALIKE). Additionally, it implements a parameter describing founder-event speciation (+J, post-colonization cladogenesis) likely important in oceanic settings^14^ and allows the comparison of different models in a statistical framework. The analyses were carried out based on the BEAST Maximum Clade Credibility (MCC) tree (Supplementary Information S5) with outgroups removed. We used the following regions in the analyses: A, Australia; B, New Caledonia; C, New Guinea; D, Vanuatu; E, Palearctic; F, Hawaii (Supplementary Information S6). The dispersal rate matrix was designed based on paleogeographic^18^,^19^ and paleoclimatic^31^ evidences (Supplementary Table S4).

### Diversification analyses

We used the program R with the BEAST MCC tree (Supplementary Information S5) from which we pruned all outgroups to investigate the diversification pattern of *Exocelina* diving beetles in a temporal framework whilst accounting for missing taxon sampling. We used different R packages to test several hypotheses regarding the evolution of *Exocelina* diving beetles in Oceania.

First, we used the package *TreePar*^55^ to estimate the potential shifts in speciation and extinction rates in the whole phylogeny through the function ‘*bd.shifts.optim’* (Supplementary Information S7). This function uses the empirical branching times from the MCC tree as an input and fits several birth-death models including 0 (constant-rate model) to several diversification rate shifts during the lineage evolution. We tested different models ranging from 0 to 5 rate shifts. All analyses were carried out with the following non-default settings: taxon sampling was set to 164/210, start = 0, end = 15 and grid = 0.1 Myr for a fine-scale estimation of rate shifts. Since New Guinea in particular might hold a large layer of unknown diversity, we also acknowledged a possible bias in clade species-richness using a total putative diversity of 300 species. We calculated AICc scores and computed Likelihood Ratio Tests (LRT) to select the best-fit between the different models allowing incrementally more shifts during the evolution of the clade.

Second, we used Bayesian Analysis of Macroevolutionary Mixtures (BAMM^56^) and its R implementation BAMMtools^57^ to identify clades with higher or lower speciation rates in the phylogeny (Supplementary Information S8). BAMM relaxes the assumption of time-homogeneous diversification that underlies alternative approaches such as the MEDUSA model^58^. It incorporates incomplete taxon sampling directly into likelihood calculations; here we assumed that our tree contains 83.0% of *Exocelina* diving beetle species. We performed multiple BAMM runs on the MCC *Exocelina* phylogeny, with 5 million generations of Markov Chain Monte Carlo (MCMC) sampling per run and sampling evolutionary parameters every 1000 generations. We reconstructed marginal distributions of net diversification rates for each branch in the BEAST MCC tree without outgroups using the posterior distribution of evolutionary parameters sampled using the reversible jump MCMC algorithm in BAMM. Finally, we reconstructed rate-through-time curves for the whole genus with or without New Guinea, but also individually for Australia, New Caledonia and New Guinea from the joint posterior density of parameters simulated with BAMM. Since it is not feasible to reconstruct plots for paraphyletic clades we considered only the large clades of Australian and New Caledonian species (including Vanuatu) therefore excluding 10 species from further calculations.

Third, we tested if diversification on the three main islands of Australia, New Caledonia and New Guinea was different throughout the evolution of the genus. To do so, we used the Multiple State Speciation Extinction (MuSSE^59^) model implemented in the *Diversitree* package^59^ (Supplementary Information S9). Three parameters for each state are included in this analysis; a speciation rate (λ), an extinction rate (μ) and a transition rate between different states (q). In order to test the hypothesis of different speciation rates between Oceanian islands, we used four states: Australia, New Caledonia, New Guinea and other (China, Hawaii, Vanuatu). The MuSSE model allows testing a wide array of parameter combinations (e.g. all speciation rates equal, all mutation rates different, some transition rates equal and some others different). We tested 36 different models and then compared their likelihood using AICc. We estimated posterior density distribution with Bayesian MCMC analyses (10,000 steps) performed with the best-fitting models and the resulting speciation, extinction and dispersal rates.

Finally, we used the package *ape*^60^ to infer lineage-through-time (LTT) plots for the New Guinean radiation as well as for the two main clades considered in the BAMM analyses using 100 pruned BEAST posterior trees.

## Additional Information

**How to cite this article**: Toussaint, E. F. A. *et al.* Mosaic patterns of diversification dynamics following the colonization of Melanesian islands. *Sci. Rep.* **5**, 16016; doi: 10.1038/srep16016 (2015).

## Supplementary Material

Supplementary Information

## Figures and Tables

**Table 1:** Genetic coverage, statistical support and divergence time estimates for the main clades of the phylogeny.

	Missing data	MrBayes PP	RAxML BS	Median ages (Ma)	95% HPD (Ma)
Root	–	–	–	97.76	65.32–134.73
Copelatinae	14.5%	1.00	100	73.50	57.02–95.21
*Lacconectus*	35.8%	1.00	100	22.81	10.87–35.27
*Copelatus*	10.2%	1.00	100	49.43	44.00–61.07
*Agaporomorphus*	5.6%	1.00	100	6.25	3.41–9.50
*Madaglymbus*	4.5%	1.00	100	7.67	3.80–11.49
*Aglymbus*	9.2%	1.00	100	8.47	4.92–13.11
*Liopterus*	41.9%	1.00	100	6.07	1.50–11.13
*Exocelina*	14.1%	1.00	99	15.35	11.20–19.92
*Exocelina* C1	9.3%	1.00	85	9.39	5.83–13.61
*Exocelina* C2	24.3%	1.00	96	12.11	8.41–16.05
*Exocelina* C3	16.8%	1.00	100	2.41	1.07–3.98
*Exocelina* C4	17.1%	0.96	92	9.29	6.79–12.34
*Exocelina* C5	37.8%	0.97	83	7.21	4.31–10.19
*Exocelina* C6	10.7%	1.00	100	5.37	3.74–7.07

Notes: PP, posterior probabilities; BS, bootstrap values.

**Table 2:** Results of the ancestral area reconstructions under different models implemented in BioGeoBEARS.

	N. param.	AIC	p-value	−logL	j	d	e	Root
DEC	2	122.7	–	59.36	0	0.0179	0.0103	A
DEC+J	3	93.94	***	43.97	0.0228	0.0000	0.0000	A
DIVALIKE	2	119.9	–	57.97	0	0.0181	0.0097	A
DIVALIKE+J	3	93.97	***	43.98	0.0227	0.0000	0.0000	A
BAYAREALIKE	2	126.3	–	61.13	0	0.0179	0.0111	A
BAYAREALIKE+J	3	98.81	***	51.40	0.0115	0.0010	0.0115	A

## References

[b1] MacArthur R. H. & Wilson E. O. An equilibrium theory of insular zoogeography. Evolution 373–387 (1963).

[b2] MacArthur R. H. & Wilson E. O. The Theory of Island Biogeography. Vol. 1 Princeton University Press (1967).

[b3] Brown J. H. & Lomolino M. V. Concluding remarks: historical perspective and the future of island biogeography theory. Global Ecol. Biogeogr. 9(1), 87–92 (2000).

[b4] Lomolino M. A call for a new paradigm of island biogeography. Global Ecol. Biogeogr. 9(1), 1–6 (2000).

[b5] Whittaker R. J., Triantis K. A. & Ladle R. J. A general dynamic theory of oceanic island biogeography: extending the MacArthur-Wilson theory to accommodate the rise and fall of volcanic islands. In: The Theory of Island Biogeography Revisited (eds Losos J. B. & Ricklefs R. E. ), pp. 88–115. Princeton University Press (2010).

[b6] Johnson K. P., Adler F. R. & Cherry J. L. Genetic and phylogenetic consequences of island biogeography. Evolution 54, 387–396 (2000). 10937215 10.1111/j.0014-3820.2000.tb00041.x

[b7] Rosindell J. & Phillimore A. B. A unified model of island biogeography sheds light on the zone of radiation. Ecol. Lett. 14(6), 552–560 (2011). 21481125 10.1111/j.1461-0248.2011.01617.x

[b8] Warren B. H. *et al.* Islands as model systems in ecology and evolution: prospects fifty years after MacArthur‐Wilson. Ecol. Lett. 18(2), 200–217 (2015). 25560682 10.1111/ele.12398

[b9] Stadler T. Recovering speciation and extinction dynamics based on phylogenies. J. Evol. Biol. 26(6), 1203–1219 (2013). 23662978 10.1111/jeb.12139

[b10] Morlon H. Phylogenetic approaches for studying diversification. Ecol. Lett. 17(4), 508–525 (2014). 24533923 10.1111/ele.12251

[b11] Ree R. H., Moore B. R., Webb C. O. & Donoghue M. J. A likelihood framework for inferring the evolution of geographic range on phylogenetic trees. Evolution 59, 2299–2311 (2005). 16396171

[b12] Ree R. H. & Smith S. A. Maximum likelihood inference of geographic range evolution by dispersal, local extinction, and cladogenesis. Syst. Biol. 57(1), 4 14 18253896 10.1080/10635150701883881

[b13] Landis M. J., Matzke N. J., Moore B. R. & Huelsenbeck J. P. Bayesian analysis of biogeography when the number of areas is large. Syst. Biol. 62(6), 789 804. 23736102 10.1093/sysbio/syt040 PMC4064008

[b14] Matzke N. J. Probabilistic historical biogeography: new models for founder-event speciation, imperfect detection, and fossils allow improved accuracy and model-testing. Front. Biogeogr. 5(4), 242–248 (2013).

[b15] Matzke N. J. Model selection in historical biogeography reveals that founder-event speciation is a crucial process in island clades. Syst. Biol. 63(6), 951–970 (2014). 25123369 10.1093/sysbio/syu056

[b16] Gillespie R. G. & Baldwin B. G. Island biogeography of remote archipelagoes. In: The theory of island biogeography revisited (eds Losos J. B. & Ricklefs R. E. ), pp. 358–387. Princeton University Press (2010).

[b17] McPeek M. A. & Brown J. M. Clade age and not diversification rate explains species richness among animal taxa. Am. Nat. 169(4), 97–106 (2007). 10.1086/512135 17427118

[b18] Hall R. Late Jurassic–Cenozoic reconstructions of the Indonesian region and the Indian Ocean. Tectonophysics 570, 1–41 (2012).

[b19] Hall R. The palaeogeography of Sundaland and Wallacea since the Late Jurassic. J. Limnol. 72(2), 1–17 (2013).

[b20] Heads M. Panbiogeography of New Caledonia, south‐west Pacific: basal angiosperms on basement terranes, ultramafic endemics inherited from volcanic island arcs and old taxa endemic to young islands. J. Biogeogr. 35(12), 2153–2175 (2008).

[b21] Sharma P. & Giribet G. A relict in New Caledonia: phylogenetic relationships of the family Troglosironidae (Opiliones: Cyphophthalmi). Cladistics 25(3), 279–294 (2009). 10.1111/j.1096-0031.2009.00252.x 34879611

[b22] Grandcolas P. *et al.* New Caledonia: a very old Darwinian island? Philos. T. Roy. Soc. B 363(1508), 3309–3317 (2008). 10.1098/rstb.2008.0122 PMC2607381 18765357

[b23] Swenson U., Nylinder S. & Munzinger J. Sapotaceae biogeography supports New Caledonia being an old Darwinian island. J. Biogeogr. 41(4), 797–809 (2014).

[b24] Neall V. E. & Trewick S. A. The age and origin of the pacific islands: a geological overview. Philos. T. Roy. Soc. B 363(1508), 3293–3308 (2008). 10.1098/rstb.2008.0119 PMC2607379 18768382

[b25] Martin H. A. Cenozoic climatic change and the development of the arid vegetation in Australia. J. Arid Environ. 66(3), 533–563 (2006).

[b26] Toussaint E. F. A. *et al.* The towering orogeny of New Guinea as a trigger for arthropod megadiversity. Nat. Comm. 5, 4001 (2014). 10.1038/ncomms5001 24874774

[b27] Balke M., Ribera I. & Vogler A. P. MtDNA phylogeny and biogeography of copelatinae, a highly diverse group of tropical diving beetles (Dytiscidae). Mol. Phylogenet. Evol. 32(3), 866–880 (2004). 15288062 10.1016/j.ympev.2004.03.014

[b28] Balke M., Pons J., Ribera I., Sagata K. & Vogler A. P. Infrequent and unidirectional colonization of hyperdiverse *Papuadytes* diving beetles in New Caledonia and New Guinea. Mol. Phylogenet. Evol. 42(2), 505–516 (2007). 16979911 10.1016/j.ympev.2006.07.019

[b29] Toussaint E. F. A. *et al.* Unveiling the diversification dynamics of Australasian predaceous diving beetles in the Cenozoic. Syst. Biol. 64(1), 3–24 (2015). 25173563 10.1093/sysbio/syu067

[b30] Shaverdo H. V., Hendrich L. & Balke M. *Exocelina baliem* sp. n., the only known pond species of New Guinea *Exocelina* Broun, 1886 (Coleoptera, Dytiscidae, Copelatinae). ZooKeys 304, 83–99 (2013). 23794909 10.3897/zookeys.304.4852 PMC3689123

[b31] Miller K. G. *et al.* The phanerozoic record of global sea-level change. Science 312, 1293–1298 (2005). 16311326 10.1126/science.1116412

[b32] Ladiges P. Y. & Cantrill D. New Caledonia–Australian connections: biogeographic patterns and geology. Aust. Syst. Bot. 20(5), 383–389 (2007).

[b33] Wewalka G., Balke M. & Hendrich L. Dytiscidae: Copelatinae (Coleoptera). In: Water beetles of New Caledonia (part 1) (eds. Jäch M. A. & Balke M. ). Monographs on Coleoptera 3, pp. 45–128. Wiener Coleopterologenverein (2010).

[b34] Monaghan M. T., Balke M., Pons J. & Vogler A. P. Beyond barcodes: complex DNA taxonomy of a South Pacific Island radiation. P. Roy. Soc. B 273(1588), 887 893 10.1098/rspb.2005.3391 PMC1560222 16618684

[b35] Abellán P., Millán A. & Ribera I. Parallel habitat‐driven differences in the phylogeographical structure of two independent lineages of Mediterranean saline water beetles. Mol. Ecol. 18(18), 3885–3902 (2009). 19702753 10.1111/j.1365-294X.2009.04319.x

[b36] Hjalmarsson A. E., Bergsten J. & Monaghan M. T. Dispersal is linked to habitat use in 59 species of water beetles (Coleoptera: Adephaga) on Madagascar. *Ecography* 10.1111/ecog.01138 (2014).

[b37] Diamond J. M. Avifauna of the eastern highlands of New Guinea, Cambridge, Nuttall Ornithological Club (1972).

[b38] Toussaint E. F. A., Sagata K., Surbakti S., Hendrich L. & Balke M. Australasian sky islands act as a diversity pump facilitating peripheral speciation and complex reversal from narrow endemic to widespread ecological supertramp. Ecol. Evol. 3(4), 1031–1049 (2013). 23610642 10.1002/ece3.517 PMC3631412

[b39] Bilton D. T., Toussaint E. F. A., Turner C. & Balke M. *Capelatus prykei* gen. n., sp. n (Coleoptera: Dytiscidae: Copelatinae) – a phylogenetically isolated diving beetle from the Western Cape of South Africa. *Syst. Entomol.* 10.1111/syen.12128 (2015).

[b40] Edgar R. C. MUSCLE: Multiple sequence alignment with high accuracy and high throughput. Nucleic Acid. Res. 32(5), 1792–1797 (2004). 15034147 10.1093/nar/gkh340 PMC390337

[b41] Lanfear R., Calcott B., Ho S. Y. & Guindon S. PartitionFinder: combined selection of partitioning schemes and substitution models for phylogenetic analyses. Mol. Biol. Evol. 29(6), 1695–1701 (2012). 22319168 10.1093/molbev/mss020

[b42] Ronquist F. *et al.* MrBayes 3.2: Efficient Bayesian phylogenetic inference and model choice across a large model space. Syst. Biol. 61(3), 539–542 (2012). 22357727 10.1093/sysbio/sys029 PMC3329765

[b43] Huelsenbeck J. P., Larget B. & Alfaro M. E. Bayesian phylogenetic model selection using reversible jump Markov chain Monte Carlo. Mol. Biol. Evol. 21(6), 1123–1133 (2004). 15034130 10.1093/molbev/msh123

[b44] Stamatakis A. RAxML-VI-HPC: maximum likelihood-based phylogenetic analyses with thousands of taxa and mixed models. Bioinformatics 22(21), 2688–2690 (2006). 16928733 10.1093/bioinformatics/btl446

[b45] Drummond A. J., Suchard M. A., Xie D. & Rambaut A. Bayesian phylogenetics with BEAUti and the BEAST 1.7. Mol. Biol. Evol. 29(8), 1969–1973 (2012). 22367748 10.1093/molbev/mss075 PMC3408070

[b46] Swofford D. L. PAUP*. Phylogenetic Analysis Using Parsimony (*and Other Methods). Version 4. Sinauer Associates, Sunderland, Massachusetts (2003).

[b47] Miller K. B. & Balke M. The unusual occurrence of aquatic beetles in amber, *Copelatus aphroditae* Balke, n. sp. and *C. predaveterus* Miller, n. sp., (Coleoptera: Dytiscidae: Copelatinae). P. Entomol. Soc. Wash. 105(4), 809–815 (2003).

[b48] Kosmowska-Ceranowicz B. & Müller C. Lithology and calcareous nannoplancton in amber-bearing Tertiary sediments from boreholes Chlapowo (Northern Poland). Bulletin of the polish academy of sciences, Earth Sci. 33, 119–128 (1985).

[b49] Ritzkowski S. K-ar-Altersbestimmungen der bernsteinführenden Sedimente des Samlandes (Paläogen, Bezirk Kaliningrad). Metalla 66, 19–23 (1997).

[b50] Engel M. S. A monograph of the baltic amber bees and evolution of the apoidea (Hymenoptera). B. Am. Mus. Nat. Hist. 1–192 (2001).

[b51] Ponomarenko A. G. New Mesozoic water beetles (Insecta, Coleoptera) from Asia. Paleontol. Zh. 2, 83–97 (1987).

[b52] Ho S. Y. W. & Phillips M. J. Accounting for calibration uncertainty in phylogenetic estimation of evolutionary divergence times. Syst. Biol. 58(3), 367–380 (2009). 20525591 10.1093/sysbio/syp035

[b53] Matzke N. J. BioGeoBEARS: BioGeography with Bayesian (and Likelihood) Evolutionary Analysis in R Scripts. University of California, Berkeley, Berkeley, CA. http://CRAN.R-project.org/package=BioGeoBEARS (2013).

[b54] Ronquist F. Dispersal-vicariance analysis: a new approach to the quantification of historical biogeography. Syst. Biol. 46(1), 195–203 (1997).

[b55] Stadler T. Inferring speciation and extinction processes from extant species data. P. Natl. Acad. Sci. USA 108(39), 16145–16146 (2011). 10.1073/pnas.1113242108 PMC3182734 21930908

[b56] Rabosky D. L., Donnellan S. C., Grundler M. & Lovette I. J. Analysis and visualization of complex macroevolutionary dynamics: an example from Australian Scincid Lizards. Syst. Biol. 63(4), 610–627 (2014). 24682412 10.1093/sysbio/syu025

[b57] Rabosky D. L. *et al.* BAMMtools: an R package for the analysis of evolutionary dynamics on phylogenetic trees. Methods Ecol. Evol. 5(7), 701–707 (2014).

[b58] Alfaro M. E. *et al.* Nine exceptional radiations plus high turnover explain species diversity in jawed vertebrates. P. Natl. Acad. Sci. USA 106(32), 13410–13414 (2009). 10.1073/pnas.0811087106 PMC2715324 19633192

[b59] FitzJohn R. G. Diversitree: comparative phylogenetic analyses of diversification in R. Methods Ecol. Evol. 5, 1084–1092 (2012).

[b60] Paradis E., Claude J. & Strimmer K. APE: analyses of phylogenetics and evolution in R language. Bioinformatics, 20(2), 289–290 (2004). 14734327 10.1093/bioinformatics/btg412

