# Supplementary material for: Mosaic patterns of diversification dynamics following the colonization of Melanesian islands
Source: Sci Rep. 2015 Nov 3;5:16016. doi: 10.1038/srep16016 (PMC4630634; doi:10.1038/srep16016)
Supplement: Supplementary Information [file srep16016-s1.doc]

**Mosaic patterns of diversification dynamics following the colonization of Melanesian islands**

Emmanuel F.A. Toussaint, Lars Hendrich, Helena Shaverdo, Michael Balke

**Supplementary Information**

**Supplementary Figure S1.** MrBayes phylogenetic relationships

**Supplementary Figure S2.** RAxML phylogenetic relationships

**Supplementary Table S1.** Results from the *TreePar* analyses conducted on the BEAST chronogram

**Supplementary Table S2.** Results from the MuSSE analyses conducted on the BEAST chronogram

**Supplementary Table S3.** List of sequenced taxa with voucher codes, locality information and habitat preference

**Supplementary Table S4.** Matrices of relative dispersal probabilities used in the BioGeoBEARS ancestral area reconstruction

**Supplementary Information S5**. BEAST chronogram in Newick format

**Supplementary Information S6**. Geography coding used to conduct the BioGeoBEARS analyses

**Supplementary Information S7**. R code used to conduct the TreePar analyses

**Supplementary Information S8**. R code used to conduct the BAMM analyses

**Supplementary Information S9**. R code used to conduct the MuSSE analyses

**Figure S1.** MrBayes phylogenetic relationships

**
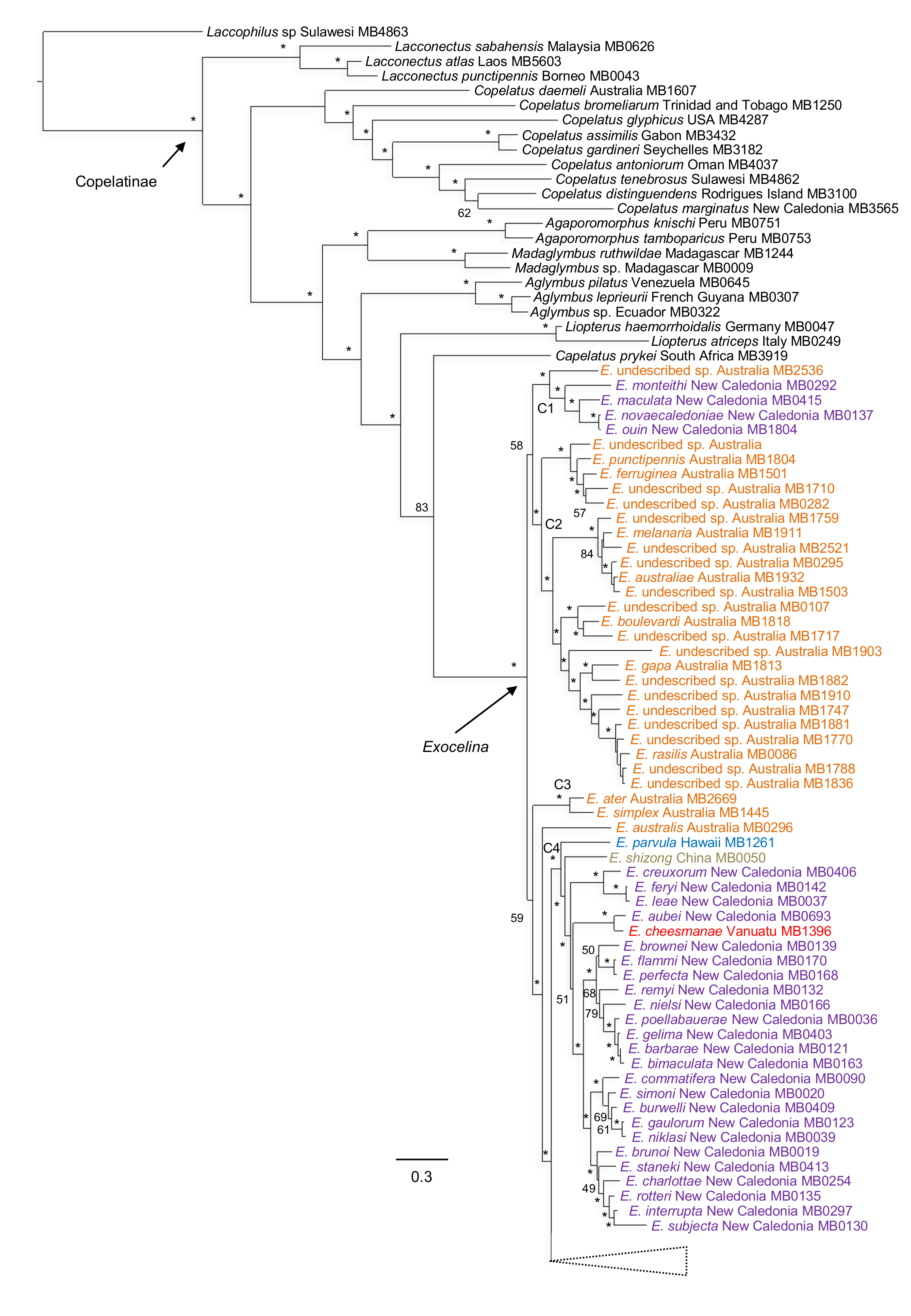
**

**
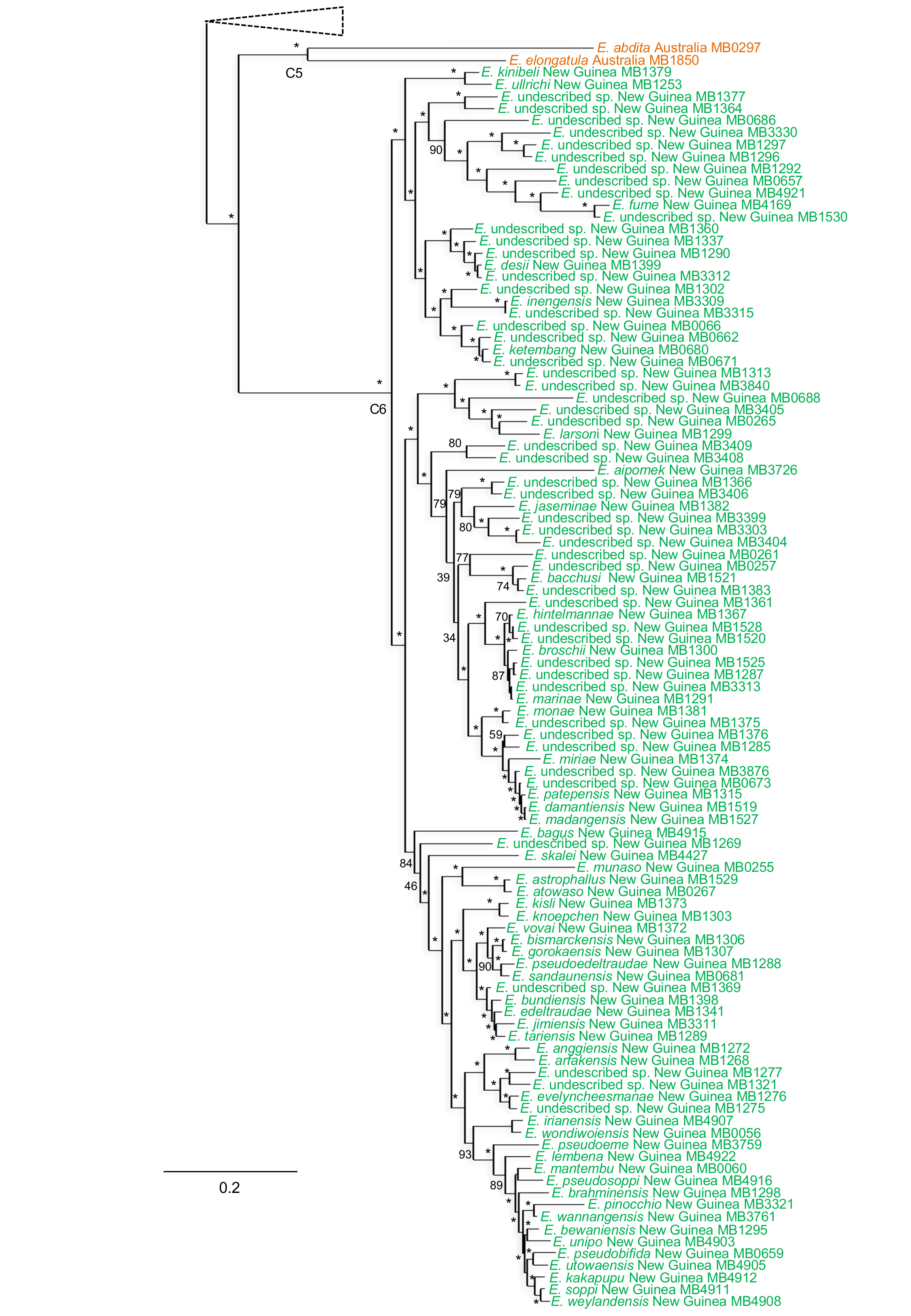
**

**Figure S2.** RAxML phylogenetic
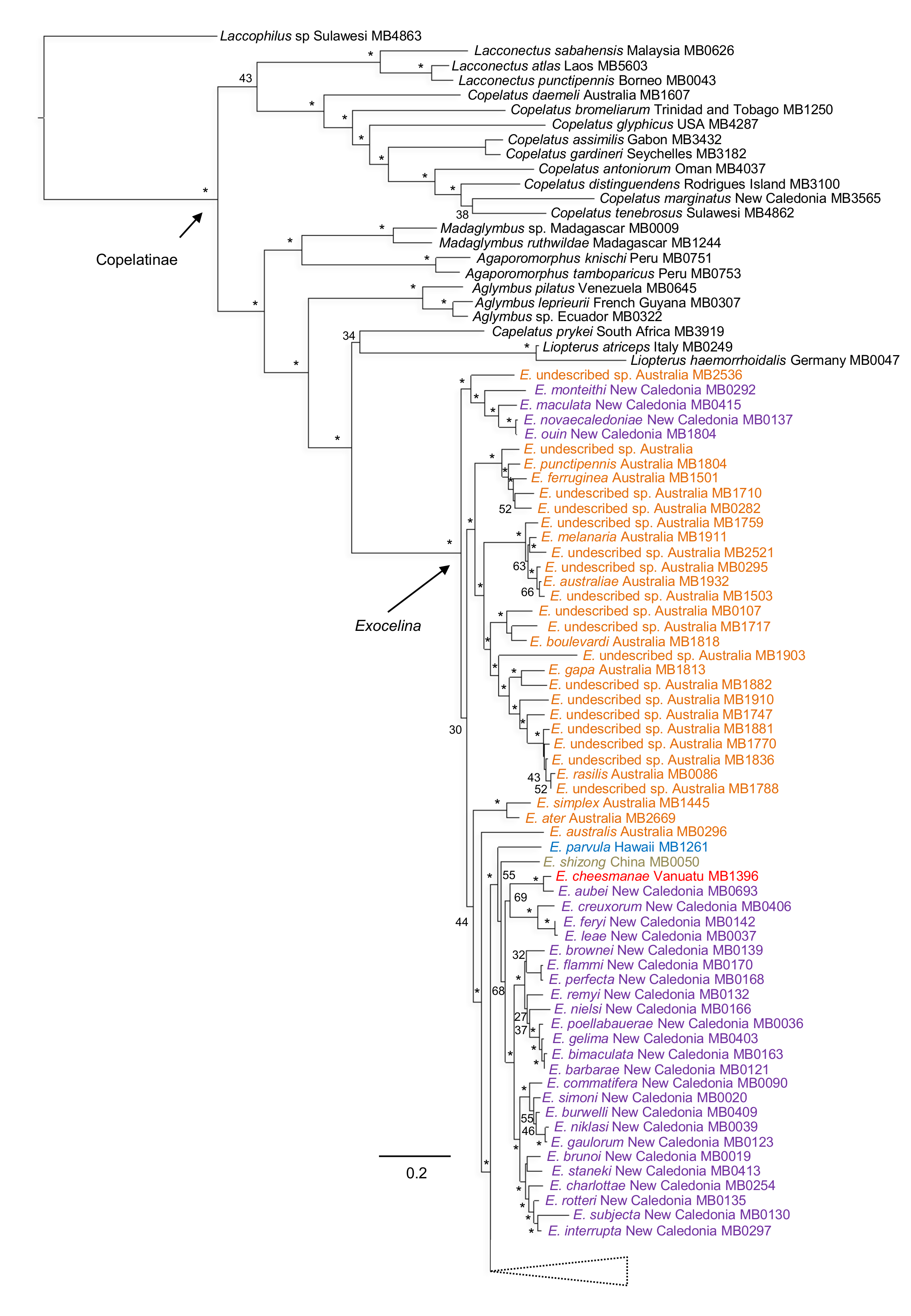
relationships


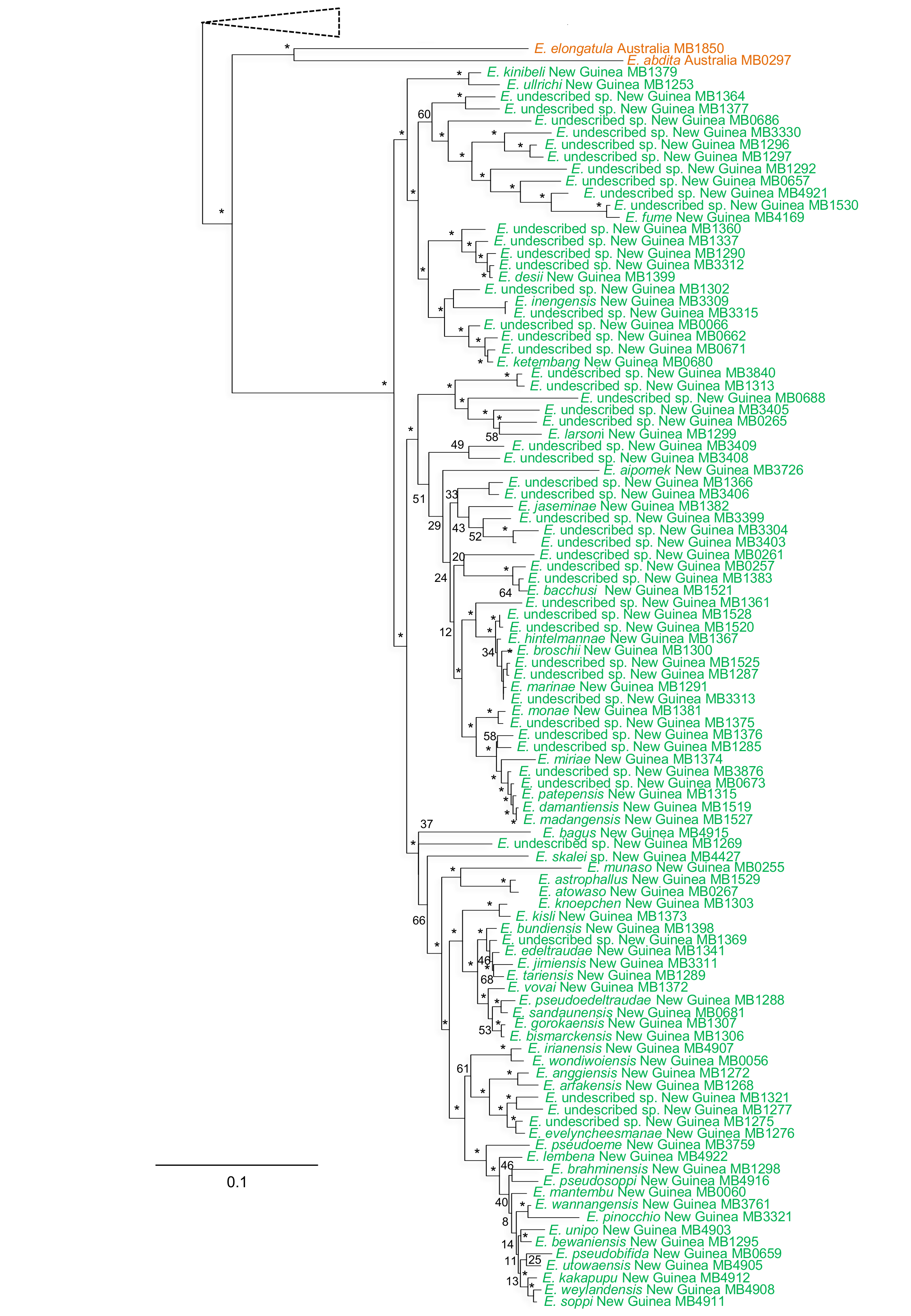


**Table S1. Results from the *TreePar* analyses conducted on the BEAST chronogram**

|  | **Pm** | **-logL** | ***P*** | **r1** | **τ1** | **st1** | **r2** | **τ2** | **st2** | **r3** | **τ3** | **st3** | **r4** | **τ4** | **st4** | **r5** | **τ 5** | **st5** | **r6** | **τ6** |
| --- | --- | --- | --- | --- | --- | --- | --- | --- | --- | --- | --- | --- | --- | --- | --- | --- | --- | --- | --- | --- |
| **SD1 no shift** | 2 | 205.701 | - | 0.270 | 0.542 | - | - | - | - | - | - | - | - | - | - | - | - | - | - | - |
| **SD1 1 shift** | **5** | **199.826** | **0.008** | **0.493** | **0.000** | **4.100** | **0.200** | **0.000** | **-** | **-** | **-** | **-** | **-** | **-** | **-** | **-** | **-** | **-** | **-** | **-** |
| **SD1 2 shifts** | 8 | 198.932 | 0.618 | 0.494 | 0.001 | 4.100 | 0.271 | 1.96 | 6.400 | 0.331 | 0.118 | - | - | - | - | - | - | - | - | - |
| **SD1 3 shifts** | 11 | 196.574 | 0.369 | 0.491 | 0.000 | 4.100 | -0.206 | 1.557 | 5.400 | 0.000 | 2.110 | 6.400 | 0.245 | 0.355 | - | - | - | - | - | - |
| **SD1 4 shifts** | 14 | 195.745 | 0.518 | 0.489 | 0.027 | 4.100 | -0.228 | 1.636 | 5.400 | 0.000 | 2.071 | 6.400 | 0.000 | 0.444 | 6.700 | 0.198 | 0.607 | - | - | - |
| **SD1 5 shifts** | 17 | 195.266 | 0.693 | 0.495 | 0.001 | 4.100 | -0.228 | 1.661 | 5.400 | -0.004 | 2.075 | 6.400 | 0.000 | 0.477 | 6.700 | 0.169 | 0.614 | 12.100 | 0.257 | 0.719 |
| **SD2 no shift** | 2 | 205.701 | - | 0.270 | 0.697 | - | - | - | - | - | - | - | - | - | - | - | - | - | - | - |
| **SD2 1 shift** | **5** | **199.609** | **0.007** | **0.544** | **0.210** | **4.100** | **0.228** | **0.000** | **-** | **-** | **-** | **-** | **-** | **-** | **-** | **-** | **-** | **-** | **-** | **-** |
| **SD2 2 shifts** | 8 | 198.034 | 0.369 | 0.705 | 0.000 | 1.500 | 0.494 | 0.289 | 4.100 | 0.171 | 0.307 | - | - | - | - | - | - | - | - | - |
| **SD2 3 shifts** | 11 | 196.429 | 0.384 | 0.709 | 0.000 | 1.500 | 0.162 | 0.393 | 1.800 | 0.457 | 0.260 | 4.100 | 0.223 | 0.072 | - | - | - | - | - | - |
| **SD2 4 shifts** | 14 | 195.310 | 0.475 | 0.687 | 0.003 | 1.500 | 0.173 | 0.357 | 1.800 | 0.414 | 0.352 | 4.100 | 0.210 | 0.518 | 5.400 | 0.220 | 0.000 | - | - | - |
| **SD2 5 shifts** | 17 | 191.615 | 0.192 | 0.716 | 0.000 | 1.500 | 0.182 | 0.381 | 1.800 | 0.445 | 0.290 | 4.100 | 0.237 | 0.416 | 5.400 | 0.000 | 0.004 | 6.400 | 0.229 | 0.297 |

Notes: SD1, analyses conducted with an estimate of diversity of 198 species; SD2, analyses conducted with an estimate of diversity of 300 species; Pm, number of parameters in the model; -LogL, the log-likelihood of the model; *P*, p-value of the Likelihood Ratio Test between the incrementally more complex models (if *P*<0.05 the model is supported). When a model is found to be significantly better than the one it is compared with then it becomes the new model to which the other models are compared with; r1, diversification rate at present; τ1, turnover rate at present; st1, most recent shift time. Other diversification and turnover rates, as well as shift times, going deeper in the past are denoted with numbers (e.g. r2, τ2, and st2). In bold is underlined the best-fit model.

**Table S2.** Results from the MuSSE analyses conducted on the BEAST chronogram

| **Model** | **Df** | **-logL** | **AIC** | **Chi²** | ***P*** |
| --- | --- | --- | --- | --- | --- |
| **minimal** | 3 | 347.49 | 700.97 | - | - |
| **all λ free** | 6 | 318.23 | 648.46 | 58.51 | 0.0000 |
| **all μ free** | 6 | 327.90 | 667.80 | 39.17 | 0.0000 |
| **all q free** | 14 | 341.05 | 710.09 | 12.88 | 0.3013 |
| **all λ and μ free** | 9 | 317.58 | 653.16 | 59.81 | 0.0000 |
| **all λ and q free** | 17 | 311.34 | 656.71 | 72.27 | 0.0000 |
| **all μ and q free** | 17 | 322.61 | 679.22 | 49.75 | 0.0000 |
| **all free** | 20 | 311.11 | 662.21 | 72.76 | 0.0000 |
| **λ1 free** | 4 | 331.00 | 670.00 | 32.97 | 0.0000 |
| **μ1 free** | 4 | 334.04 | 676.08 | 26.89 | 0.0000 |
| **q1 free** | 9 | 346.05 | 710.09 | 2.88 | 0.8236 |
| **λ1 and μ1 free** | 5 | 330.74 | 671.47 | 33.50 | 0.0000 |
| **λ1 and q1 free** | 10 | 328.83 | 677.66 | 37.32 | 0.0000 |
| **μ1 and q1 free** | 10 | 331.88 | 683.76 | 31.21 | 0.0000 |
| **λ1, μ1 and q1 free** | 11 | NA | NA | NA | NA |
| **λ2 free** | 4 | 323.65 | 655.30 | 47.67 | 0.0000 |
| **μ2 free** | 4 | 329.97 | 667.95 | 35.02 | 0.0000 |
| **q2 free** | 9 | 344.06 | 706.12 | 6.86 | 0.3342 |
| **λ2 and μ2 free** | 5 | 323.25 | 656.49 | 48.48 | 0.0000 |
| **λ2 and q2 free** | 10 | 320.31 | 660.61 | 54.36 | 0.0000 |
| **μ2 and q2 free** | 10 | 326.56 | 673.12 | 41.85 | 0.0000 |
| **λ2, μ2 and q2 free** | 11 | 320.01 | 662.02 | 54.95 | 0.0000 |
| **λ3 free** | 4 | 347.41 | 702.83 | 0.14 | 0.7039 |
| **μ3 free** | 4 | 347.38 | 702.77 | 0.20 | 0.6523 |
| **q3 free** | 9 | 344.80 | 707.60 | 5.37 | 0.4971 |
| **λ3 and μ3 free** | 5 | 345.22 | 700.43 | 4.54 | 0.1031 |
| **λ3 and q3 free** | 10 | 341.59 | 703.18 | 11.79 | 0.1075 |
| **μ3 and q3 free** | 10 | 342.64 | 705.28 | 9.69 | 0.2069 |
| **λ3, μ3 and q3 free** | 11 | 341.58 | 705.15 | 11.81 | 0.1597 |
| **λ4 free** | 4 | 345.46 | 698.93 | 4.05 | 0.0443 |
| **μ4 free** | 4 | 346.57 | 701.13 | 1.84 | 0.1752 |
| **q4 free** | 9 | 342.81 | 703.63 | 9.35 | 0.1550 |
| **λ4 and μ4 free** | 5 | 342.69 | 695.39 | 9.58 | 0.0083 |
| **λ4 and q4 free** | 10 | 339.05 | 698.11 | 16.87 | 0.0183 |
| **μ4 and q4 free** | 10 | 326.40 | 672.81 | 42.17 | 0.0000 |
| **λ4, μ4 and q4 free** | 11 | 326.34 | 674.69 | 42.28 | 0.0000 |

Notes: Notes: Df, degree of freedom of each model; -LogL, the log-likelihood of the model; AIC, the Akaike information criterion; Chi^2^, the chi-squared distribution compared to the minimal model; and *P*, the p-value (if *P*<0.05 the model is supported); λ, the speciation rate; μ, the extinction rate; q, the transition rate between two states.

**Table S3.** List of sequenced taxa with voucher codes, locality information and habitat preference.

| Genus | Species | ID | Country | Province | Locality | Habitat |
| --- | --- | --- | --- | --- | --- | --- |
| *Agaporomorphus* | *knischi* | MB0751 | Peru | Madre de Dios | Tambopata | - |
| *Agaporomorphus* | *tambopatensis* | MB0753 | Peru | Madre de Dios | Tambopata | - |
| *Aglymbus* | *leprieurii* | MB0307 | France | French Guyana | Petit Saut | - |
| *Aglymbus* | *pilatus* | MB0645 | Venezuela | Amazonas | Tobogan de la Selva | - |
| *Aglymbus* | sp | MB0322 | Ecuador | Orellana | Tiputini | - |
| *Capelatus* | *prykei* | MB3919 | South Africa | Western Cape | Cape Peninsula | - |
| *Copelatus* | *antoniorum* | MB4037 | Oman | Al Batinah | Wadi Bani Khalid | - |
| *Copelatus* | *assimilis* | MB3432 | Gabon | Haut Ogooué | Plateaux Batéké | - |
| *Copelatus* | *bromeliarum* | MB1250 | Trinidad and Tobago | Trinidad | El Tucuche | - |
| *Copelatus* | *daemeli* | MB1607 | Australia | Northern Territory | Greenant Creek | - |
| *Copelatus* | *distinguendens* | MB3100 | Mauritius | Rodrigues Island | Solitude | - |
| *Copelatus* | *gardineri* | MB3182 | Seychelles | - | - | - |
| *Copelatus* | *glyphicus* | MB4287 | United States of America | California | Yolo County | - |
| *Copelatus* | *marginatus* | MB3565 | New Caledonia | Grande Terre | La Foa | - |
| *Copelatus* | *tenebrosus* | MB4862 | Indonesia | Sulawesi | Batudaka Island | - |
| *Exocelina* | *abditus* | MB0297 | Australia | Northern Territory | Newhaven Sanctuary | Riparian |
| *Exocelina* | *aipomek* | MB3726 | Papua New Guinea | Sandaun | Ofektamin | Riparian |
| *Exocelina* | *anggiensis* | MB1272 | Indonesia | West Papua | Arfak Mts. - Anggi | Riparian |
| *Exocelina* | *arfakensis* | MB1268 | Indonesia | West Papua | Arfak Mts.: Mokwam | Riparian |
| *Exocelina* | *astrophallus* | MB1529 | Papua New Guinea | Madang | Adalbert Range | Riparian |
| *Exocelina* | *atowaso* | MB0267 | Papua New Guinea | Madang | Bundi | Riparian |
| *Exocelina* | *ater* | MB2669 | Australia | Western Australia | D’Entrecastaux | Lentic |
| *Exocelina* | *aubei* | MB0693 | New Caledonia | Ile des Pins | - | Riparian |
| *Exocelina* | *australiae* | MB1932 | Australia | New South Wales | Mt. Kosciusko | Riparian |
| *Exocelina* | *australis* | MB0296 | Australia | Southern Australia | Flinders range | Riparian |
| *Exocelina* | *bacchusi* | MB1521 | Papua New Guinea | Eastern Highlands | Akameku – Brahmin, Bismarck range | Riparian |
| *Exocelina* | *bagus* | MB4915 | Indonesia | Papua | Road Nabire Ilaga KM55 | Riparian |
| *Exocelina* | *barbarae* | MB0121 | New Caledonia | Grande Terre | Aoupinié | Riparian |
| *Exocelina* | *bewaniensis* | MB1295 | Papua New Guinea | Sandaun | Bewani Mts. | Riparian |
| *Exocelina* | *bimaculata* | MB0163 | New Caledonia | Grande Terre | Ouégoa | Riparian |
| *Exocelina* | *bismarckensis* | MB1306 | Papua New Guinea | Eastern Highlands | Daulo | Riparian |
| *Exocelina* | *boulevardi* | MB1818 | Australia | New South Wales | Imlay Road | Lentic |
| *Exocelina* | *brahminensis* | MB1298 | Papua New Guinea | Madang | Adalbert Range | Riparian |
| *Exocelina* | *broschii* | MB1525 | Papua New Guinea | Enga | Wabag | Riparian |
| *Exocelina* | *brownei* | MB0139 | New Caledonia | Grande Terre | Thio | Riparian |
| *Exocelina* | *brunoi* | MB0019 | New Caledonia | Grande Terre | Mt. Canala | Riparian |
| *Exocelina* | *bundiensis* | MB1398 | Papua New Guinea | Eastern Highlands | Bismarck Range | Riparian |
| *Exocelina* | *burwelli* | MB0409 | New Caledonia | Grande Terre | Gelima | Riparian |
| *Exocelina* | *charlottae* | MB0254 | New Caledonia | Grande Terre | Mt. Koghis | Riparian |
| *Exocelina* | *cheesmanae* | MB1396 | Vanuatu | Espiritu Santo | Penarou | Riparian |
| *Exocelina* | *commatifera* | MB0090 | New Caledonia | Grande Terre | Mt. Panié | Riparian |
| *Exocelina* | *creuxorum* | MB0406 | New Caledonia | Grande Terre | Pic d’Amoa | Riparian |
| *Exocelina* | *damantiensis* | MB1519 | Papua New Guinea | Eastern Highlands | Bismarck Range: Akameku-Brahmin | Riparian |
| *Exocelina* | *desii* | MB1399 | Papua New Guinea | Eastern Highlands | Bismarck Range | Riparian |
| *Exocelina* | *edeltraudae* | MB1341 | Papua New Guinea | Western Highlands | Kurumul | Riparian |
| *Exocelina* | *elongatula* | MB1850 | Australia | New South Wales | Chevalley Lane | Lentic |
| *Exocelina* | *evelyncheesmanae* | MB1276 | Papua New Guinea | West Papua | Waigeo – Mt. Nok | Riparian |
| *Exocelina* | *ferruginea* | MB1501 | Australia | Southern Australia | Penola | Lentic |
| *Exocelina* | *feryi* | MB0142 | New Caledonia | Grande Terre | Rivière Bleue | Riparian |
| *Exocelina* | *flammi* | MB0170 | New Caledonia | Grande Terre | Ouégoa | Riparian |
| *Exocelina* | *fume* | MB4169 | Papua New Guinea | Madang | Wannang | Riparian |
| *Exocelina* | *gapa* | MB1813 | Australia | Queensland | Cunninghams Gap | Riparian |
| *Exocelina* | *gaulorum* | MB0123 | New Caledonia | Grande Terre | Aoupinié | Riparian |
| *Exocelina* | *gelima* | MB0403 | New Caledonia | Grande Terre | Gelima | Riparian |
| *Exocelina* | *gorokaensis* | MB1307 | Papua New Guinea | Eastern Highlands | Daulo | Riparian |
| *Exocelina* | *hintelmannae* | MB1367 | Papua New Guinea | Gulf | Marawaka | Riparian |
| *Exocelina* | *inengensis* | MB3309 | Papua New Guinea | Western Highlands | Simbai | Riparian |
| *Exocelina* | *interrupta* | MB0253 | New Caledonia | Grande Terre | Mt. Mou | Riparian |
| *Exocelina* | *irianensis* | MB4907 | Indonesia | Papua | Road Nabire Ilaga KM55 | Riparian |
| *Exocelina* | *jaseminae* | MB1382 | Papua New Guinea | Morobe | Herzog Mts | Riparian |
| *Exocelina* | *jimiensis* | MB3311 | Papua New Guinea | Western Highlands | Schrader Range, Simbai-Jimi | Riparian |
| *Exocelina* | *kakapupu* | MB4912 | Indonesia | Papua | Road Nabire Ilaga KM60 | Riparian |
| *Exocelina* | *ketembang* | MB0680 | Papua New Guinea | Sandaun | Mekil | Riparian |
| *Exocelina* | *kinibeli* | MB1379 | Papua New Guinea | Morobe | Menyamya: Mt. Inji | Riparian |
| *Exocelina* | *kisli* | MB1373 | Papua New Guinea | Gulf | Ande-Menyamya | Riparian |
| *Exocelina* | *knoepfchen* | MB1303 | Papua New Guinea | Eastern Highlands | Onerunka | Riparian |
| *Exocelina* | *larsoni* | MB1299 | Papua New Guinea | Madang | Adalbert Range | Riparian |
| *Exocelina* | *leae* | MB0037 | New Caledonia | Grande Terre | Aoupinié | Riparian |
| *Exocelina* | *lembena* | MB4922 | Papua New Guinea | East Sepik | Lembena | Riparian |
| *Exocelina* | *maculata* | MB0415 | New Caledonia | Grande Terre | Mt. Humboldt | Riparian |
| *Exocelina* | *madangensis* | MB1527 | Papua New Guinea | Enga | Wapanamanda | Riparian |
| *Exocelina* | *mantembu* | MB0060 | Indonesia | Japen Island | Mantembu | Riparian |
| *Exocelina* | *marinae* | MB1291 | Papua New Guinea | Southern Highlands | Tari-Koroba | Riparian |
| *Exocelina* | *melanaria* | MB1911 | Australia | Queensland | Maryborough | Riparian |
| *Exocelina* | *miriae* | MB1374 | Papua New Guinea | Gulf | Menyamya: Mt. Inji | Riparian |
| *Exocelina* | *monae* | MB1381 | Papua New Guinea | Morobe | Herzog Mts. | Riparian |
| *Exocelina* | *monteithi* | MB0292 | New Caledonia | Grande Terre | Mt. Panié | Riparian |
| *Exocelina* | *munaso* | MB0255 | Papua New Guinea | Simbu / Eastern Highlands | Wara Sera | Riparian |
| *Exocelina* | *nielsi* | MB0166 | New Caledonia | Grande Terre | Dumbea | Riparian |
| *Exocelina* | *niklasi* | MB0039 | New Caledonia | Grande Terre | Aoupinié | Riparian |
| *Exocelina* | *novaecaledoniae* | MB0137 | New Caledonia | Grande Terre | Mt. Humboldt | Riparian |
| *Exocelina* | *ouin* | MB0410 | New Caledonia | Grande Terre | Mt. Quinn | Riparian |
| *Exocelina* | *parvula* | MB1261 | United States of America | Hawaii | Kauai Island | Riparian |
| *Exocelina* | *patepensis* | MB1315 | Papua New Guinea | Morobe | Kwapsanek | Riparian |
| *Exocelina* | *perfecta* | MB0168 | New Caledonia | Grande Terre | Camp Minier | Riparian |
| *Exocelina* | *pinocchio* | MB3321 | Papua New Guinea | Madang | Usino | Riparian |
| *Exocelina* | *poellabauerae* | MB0036 | New Caledonia | Grande Terre | Mt. Panié | Riparian |
| *Exocelina* | *pseudobifida* | MB0659 | Papua New Guinea | Sandaun | Mekil | Riparian |
| *Exocelina* | *pseudoedeltraudae* | MB1288 | Papua New Guinea | Hela | S Tari, Hides Gas | Riparian |
| *Exocelina* | *pseudoeme* | MB3759 | Papua New Guinea | Sandaun | Mianmin | Riparian |
| *Exocelina* | *pseudosoppi* | MB4916 | Indonesia | Papua | road Nabire Ilaga KM62 | Riparian |
| *Exocelina* | *punctipennis* | MB1804 | Australia | New South Wales | Braidwood | Lentic |
| *Exocelina* | *rasilis* | MB0086 | Australia | Queensland | Cunninghams Gap | Riparian |
| *Exocelina* | *remyi* | MB0132 | New Caledonia | Grande Terre | Mt. Mou | Riparian |
| *Exocelina* | *rotteri* | MB0135 | New Caledonia | Grande Terre | Thio | Riparian |
| *Exocelina* | *sandaunensis* | MB0681 | Papua New Guinea | Sandaun | Sandaun, Mekil | Riparian |
| *Exocelina* | *shizong* | MB0050 | China | Yunnan | Shizong | Riparian |
| *Exocelina* | *simoni* | MB0020 | New Caledonia | Grande Terre | Mt. Canala | Riparian |
| *Exocelina* | *simplex* | MB1445 | Australia | Southern Australia | Penola | Lentic |
| *Exocelina* | *skalei* | MB4427 | Indonesia | West Papua | Kaimana: Triton bay, Kamaka village | Riparian |
| *Exocelina* | *soppi* | MB4911 | Indonesia | Papua | Road Nabire Ilaga KM52 | Riparian |
| *Exocelina* | *staneki* | MB0413 | New Caledonia | Grande Terre | Mt. Quinn | Riparian |
| *Exocelina* | *subjecta* | MB0130 | New Caledonia | Grande Terre | Mt. Mou | Riparian |
| *Exocelina* | *tariensis* | MB1289 | Papua New Guinea | Southern Highlands | Mt. Ambua | Riparian |
| *Exocelina* | *ullrichi* | MB1253 | Papua New Guinea | Eastern Highlands | Wapi Creek | Riparian |
| *Exocelina* | *unipo* | MB4903 | Indonesia | Papua | Road Nabire Ilaga KM108 | Riparian |
| *Exocelina* | *utowaensis* | MB4905 | Indonesia | Papua | road Nabire Ilaga KM62 | Riparian |
| *Exocelina* | *vovai* | MB1372 | Papua New Guinea | Morobe | Menyamya-Aseki | Riparian |
| *Exocelina* | *wannangensis* | MB3761 | Papua New Guinea | Madang | Wannang | Riparian |
| *Exocelina* | *weylandensis* | MB4908 | Indonesia | Papua | Road Nabire Ilaga KM60 | Riparian |
| *Exocelina* | *wondiwoiensis* | MB0056 | Indonesia | West Papua | Wasior | Riparian |
| *Exocelina* | undescribed sp. | - | Australia | Northern Territory | Holmes Jungle Reserve | Lentic |
| *Exocelina* | undescribed sp. | MB0066 | Indonesia | Papua | N Wamena rd to Passvalley | Riparian |
| *Exocelina* | undescribed sp. | MB0107 | Australia | Southern Australia | Penola | Lentic |
| *Exocelina* | undescribed sp. | MB0257 | Papua New Guinea | Simbu | Crater Mountain | Riparian |
| *Exocelina* | undescribed sp. | MB0261 | Papua New Guinea | Simbu | Crater Mountain | Riparian |
| *Exocelina* | undescribed sp. | MB0265 | Papua New Guinea | Simbu | Crater Mountain | Riparian |
| *Exocelina* | undescribed sp. | MB0282 | Australia | Western Australia | Perenjoi | Lentic |
| *Exocelina* | undescribed sp. | MB0295 | Australia | Tasmania | Terraleah | Riparian |
| *Exocelina* | undescribed sp. | MB0657 | Papua New Guinea | Sandaun | Sokamin | Riparian |
| *Exocelina* | undescribed sp. | MB0662 | Papua New Guinea | Sandaun | May River | Riparian |
| *Exocelina* | undescribed sp. | MB0671 | Papua New Guinea | Sandaun | Mekil | Riparian |
| *Exocelina* | undescribed sp. | MB0673 | Papua New Guinea | Sandaun | Mianmin | Riparian |
| *Exocelina* | undescribed sp. | MB0686 | Papua New Guinea | Sandaun | Mekil | Riparian |
| *Exocelina* | undescribed sp. | MB0688 | Papua New Guinea | Sandaun | May River | Riparian |
| *Exocelina* | undescribed sp. | MB1269 | Indonesia | West Papua | Ransiki-Anggi | Riparian |
| *Exocelina* | undescribed sp. | MB1275 | Indonesia | West Papua | Waigeo – Mt. Nok | Riparian |
| *Exocelina* | undescribed sp. | MB1277 | Indonesia | West Papua | Batanta Utara | Riparian |
| *Exocelina* | undescribed sp. | MB1285 | Papua New Guinea | Morobe | Huon, Kabwum | Riparian |
| *Exocelina* | undescribed sp. | MB1287 | Papua New Guinea | Southern Highlands | Tari | Riparian |
| *Exocelina* | undescribed sp. | MB1290 | Papua New Guinea | Southern Province | Mt. Ambua | Riparian |
| *Exocelina* | undescribed sp. | MB1292 | Papua New Guinea | Southern Province | Koroba | Riparian |
| *Exocelina* | undescribed sp. | MB1296 | Papua New Guinea | Sandaun | Bewani Mts. | Riparian |
| *Exocelina* | undescribed sp. | MB1297 | Papua New Guinea | Madang | Adalbert Range | Riparian |
| *Exocelina* | undescribed sp. | MB1300 | Papua New Guinea | Madang | Adalbert Mts., Keki | Riparian |
| *Exocelina* | undescribed sp. | MB1302 | Papua New Guinea | Eastern Highlands | Kainantu | Riparian |
| *Exocelina* | undescribed sp. | MB1313 | Papua New Guinea | Morobe | Kwapsanek | Riparian |
| *Exocelina* | undescribed sp. | MB1321 | Indonesia | West Papua | Fak Fak | Riparian |
| *Exocelina* | undescribed sp. | MB1337 | Papua New Guinea | Southern Highlands | Sopukul | Riparian |
| *Exocelina* | undescribed sp. | MB1360 | Papua New Guinea | Enga | Kumul Lodge | Riparian |
| *Exocelina* | undescribed sp. | MB1361 | Papua New Guinea | Gulf | Marawaka: Andakombe towards Morobe | Riparian |
| *Exocelina* | undescribed sp. | MB1364 | Papua New Guinea | Madang | Bismarck Range: Akameku-Brahmin | Riparian |
| *Exocelina* | undescribed sp. | MB1366 | Papua New Guinea | Eastern Highlands | Ande | Riparian |
| *Exocelina* | undescribed sp. | MB1369 | Papua New Guinea | Gulf | Andakombe towards Morobe | Riparian |
| *Exocelina* | undescribed sp. | MB1375 | Papua New Guinea | Gulf | Menyamya: Mt. Inji | Riparian |
| *Exocelina* | undescribed sp. | MB1376 | Papua New Guinea | Morobe | Menyamya | Riparian |
| *Exocelina* | undescribed sp. | MB1377 | Papua New Guinea | Morobe | Menyamya: Mt. Inji | Riparian |
| *Exocelina* | undescribed sp. | MB1383 | Papua New Guinea | Morobe | Herzog Mts., Wagau | Riparian |
| *Exocelina* | undescribed sp. | MB1503 | Australia | Southern Australia | Forreston Watts Gully | Riparian |
| *Exocelina* | undescribed sp. | MB1520 | Papua New Guinea | Eastern Highlands | Bismarck Range: Akameku-Brahmin | Riparian |
| *Exocelina* | undescribed sp. | MB1528 | Papua New Guinea | Western Highlands | Mt. Hagen | Riparian |
| *Exocelina* | undescribed sp. | MB1530 | Papua New Guinea | Madang | Adalbert Range | Riparian |
| *Exocelina* | undescribed sp. | MB1710 | Australia | Western Australia | Midlands | Lentic |
| *Exocelina* | undescribed sp. | MB1717 | Australia | Tasmania | Geeveston | Lentic |
| *Exocelina* | undescribed sp. | MB1747 | Australia | Queensland | Kuranda | Riparian |
| *Exocelina* | undescribed sp. | MB1759 | Australia | Queensland | Lake Kinchant | Riparian |
| *Exocelina* | undescribed sp. | MB1770 | Australia | Queensland | Lake Kinchant | Riparian |
| *Exocelina* | undescribed sp. | MB1788 | Australia | New South Wales | Braidwood road | Riparian |
| *Exocelina* | undescribed sp. | MB1836 | Australia | New South Wales | Crooked Creek | Riparian |
| *Exocelina* | undescribed sp. | MB1881 | Australia | Queensland | Wolvi | Riparian |
| *Exocelina* | undescribed sp. | MB1882 | Australia | Queensland | Cape Tribulation road | Riparian |
| *Exocelina* | undescribed sp. | MB1903 | Australia | Queensland | Manorina | Riparian |
| *Exocelina* | undescribed sp. | MB1910 | Australia | Queensland | Cape Tribulation road | Riparian |
| *Exocelina* | undescribed sp. | MB2521 | Australia | Queensland | Wolvi | Riparian |
| *Exocelina* | undescribed sp. | MB2536 | Australia | New South Wales | Bellingen | Riparian |
| *Exocelina* | undescribed sp. | MB3303 | Papua New Guinea | NCD | Varirata | Riparian |
| *Exocelina* | undescribed sp. | MB3312 | Papua New Guinea | Western Highlands | Simbai: Gonzsidai - Sarup | Riparian |
| *Exocelina* | undescribed sp. | MB3313 | Papua New Guinea | Western Highlands | Gonzsidai-Sarup | Riparian |
| *Exocelina* | undescribed sp. | MB3315 | Papua New Guinea | Western Highlands | Simbai: Jimi valley, above Sendiap Station | Riparian |
| *Exocelina* | undescribed sp. | MB3330 | Indonesia | Papua | Cyclops Mts.: Doyo | Riparian |
| *Exocelina* | undescribed sp. | MB3399 | Papua New Guinea | Central | Woitape | Riparian |
| *Exocelina* | undescribed sp. | MB3404 | Papua New Guinea | Central | Kokoda Trail | Riparian |
| *Exocelina* | undescribed sp. | MB3405 | Papua New Guinea | Central | Kokoda Trail | Riparian |
| *Exocelina* | undescribed sp. | MB3406 | Papua New Guinea | Central | Myola | Riparian |
| *Exocelina* | undescribed sp. | MB3408 | Papua New Guinea | Central | Myola | Riparian |
| *Exocelina* | undescribed sp. | MB3409 | Papua New Guinea | Central | Myola | Riparian |
| *Exocelina* | undescribed sp. | MB3840 | Papua New Guinea | Morobe | Garaina | Riparian |
| *Exocelina* | undescribed sp. | MB3876 | Papua New Guinea | Morobe | Garaina | Riparian |
| *Exocelina* | undescribed sp. | MB4921 | Papua New Guinea | East Sepik | Lembena | Riparian |
| *Lacconectus* | *atlas* | MB5602 | Laos | Attapeu | Annam Highlands Mts. | - |
| *Lacconectus* | *punctipennis* | MB0043 | Indonesia | Borneo | Betung Karihum | - |
| *Lacconectus* | *sabahensis* | MB0626 | Malaysia | Sabah | - | - |
| Laccophilus | sp | MB4863 | Indonesia | Sulawesi | Batudaka Island | - |
| Liopterus | atriceps | MB0249 | Italy | Sardinia | - | - |
| *Liopterus* | *haemorrhoidalis* | MB0047 | Germany | Bavaria | Benediktbeuern, Kochelsee | - |
| *Madaglymbus* | *ruthwildae* | MB1244 | Madagascar | Antsiranana | Ankarana | - |
| *Madaglymbus* | sp | MB0009 | Madagascar | Antananarivo | Anjozorobe | - |

Notes: ID, extraction number of the specimen selected for the study.

**Table S4.** Matrices of relative dispersal probabilities used in the BioGeoBEARS ancestral area reconstruction

|  | **Australia** | **New Caledonia** | **New Guinea** | **Vanuatu** | **China** | **Hawaii** |
| --- | --- | --- | --- | --- | --- | --- |
| **Australia** | - | 0.1 | 0.75 | 0.01 | 0.01 | 0.01 |
| **New Caledonia** | 0.1 | - | 0.1 | 0.25 | 0.01 | 0.01 |
| **New Guinea** | 0.75 | 0.1 | - | 0.01 | 0.01 | 0.01 |
| **Vanuatu** | 0.01 | 0.25 | 0.01 | - | 0.01 | 0.01 |
| **China** | 0.01 | 0.01 | 0.01 | 0.01 | - | 0.01 |
| **Hawaii** | 0.01 | 0.01 | 0.01 | 0.01 | 0.01 | - |

**Supplementary Information S5**. BEAST chronogram in Newick format

(Laccophilus_sp_Sulawesi_MB4863:97.76453998904333,((Lacconectus_sabahensis_Malaysia_MB0626:22.812162127528016,(Lacconectus_atlas_Laos_MB5602:3.3030612786319278,Lacconectus_punctipennis_Borneo_MB0043:3.3030612786319278):19.509100848895994):50.688333809502936,((Copelatus_daemeli_Australia_MB1607:49.43271263818567,(Copelatus_bromeliarum_Trinidad_and_Tobago_MB1250:40.0599393652555,(Copelatus_glyphicus_United_States_America_MB4287:35.88161863484244,((Copelatus_assimilis_Gabon_MB3432:3.595559840591095,Copelatus_gardineri_Seychelles_MB3182:3.595559840591095):27.95618172651008,(Copelatus_antoniorum_Oman_MB4037:22.048067567698084,(Copelatus_tenebrosus_Sulawesi_MB4862:16.870897207918016,(Copelatus_distinguendens_Rodrigues_Island_MB3100:15.199095339310738,Copelatus_marginatus_New_Caledonia_MB3565:15.199095339310738):1.671801868607261):5.177170359780206):9.503673999403123):4.329877067741133):4.178320730413205):9.37277327293009):20.463441206377695,(((Agaporomorphus_knischi_Peru_MB0751:6.245566399743145,Agaporomorphus_tamboparicus_Peru_MB0753:6.245566399743145):34.77306106225746,(Madaglymbus_ruthwildae_Madagascar_MB1244:7.672047375876613,Madaglymbus_sp_Madagascar_MB0009:7.672047375876613):33.34658008612408):13.799113835205489,((Aglymbus_pilatus_Venezuela_MB0645:8.469769739003187,(Aglymbus_leprieurii_French_Guyana_MB0307:2.662482974093457,Aglymbus_sp_Ecuador_MB0322:2.662482974093457):5.807286764909745):36.214478405975335,((Liopterus_atriceps_Italy_MB0047:6.068961946100681,Liopterus_haemorrhoidalis_Germany_MB0249:6.068961946100681):29.08065921902875,(Capelatus_prykei_South_Africa_MB3919:30.560995368325123,(((Exocelina_sp_nov_10_Australia_MB2536:9.385476360745892,(Exocelina_monteithi_New_Caledonia_MB0292:4.359670584716556,(Exocelina_maculata_New_Caledonia_MB0415:2.1561171535801638,(Exocelina_novaecaledoniae_New_Caledonia_MB0137:0.4798502426818206,Exocelina_ouin_New_Caledonia_MB0410:0.4798502426818206):1.676266910898349):2.2035534311363887):5.025805776029369):4.945927600238946,((Exocelina_stygobiont_sp_rasjadi_Australia:5.371492798444813,(Exocelina_punctipennis_Australia_1804:3.3153124506793876,(Exocelina_ferrugineus_Australia_MB1501:2.364468184445649,(Exocelina_sp_nov_5_Australia_MB1710:1.8965895310657133,Exocelina_sp_nov_6_Australia_MB282:1.8965895310657133):0.46787865337996193):0.9508442662337435):2.0561803477654403):6.739164705532412,((Exocelina_sp_nov_4_Australia_MB1759:2.758190272681075,((Exocelina_melanaria_Australia_MB1911:1.4665707018338956,Exocelina_sp_nov_3_Australia_MB2521:1.4665707018338956):0.729154509077417,(Exocelina_sp_nov_1_Australia_MB295:0.8672808941121658,(Exocelina_australiae_Australia_MB1932:0.5499198420221313,Exocelina_sp_nov_2_Australia_MB1503:0.5499198420221313):0.31736105209003207):1.328444316799175):0.5624650617697635):7.181965726376561,((Exocelina_sp_nov_8_Australia_MB107:3.8811259330866923,(Exocelina_boulvardi_Australia_MB1818:2.8844309352878073,Exocelina_sp_nov_7_Australia_MB1717:2.8844309352878073):0.9966949977988775):4.372090915741241,(Exocelina_sp_nov_12_Australia_MB1903:6.778703792346414,((Exocelina_gapa_Australia_MB1813:3.561185783737931,Exocelina_sp_nov_11_Australia_MB1882:3.561185783737931):1.5073228439530881,(Exocelina_sp_nov_18_Australia_MB1910:3.686901570385938,(Exocelina_sp_nov_17_Australia_MB1747:2.4338052688342464,(Exocelina_sp_nov_15_glyptus_Australia_MB1881:1.3861503944956397,(Exocelina_sp_nov_14_glyptus_Australia_MB1770:1.0430252263799442,(Exocelina_rasilis_Australia_MB0086:0.6053826148001925,(Exocelina_sp_nov_13_glyptus_Australia_MB1788:0.3305570360706582,Exocelina_sp_nov_16_glyptus_Australia_MB1836:0.3305570360706582):0.27482557872950564):0.43764261157977724):0.34312516811569505):1.047654874338581):1.253096301551691):1.3816070573050818):1.7101951646553903):1.4745130564815518):1.6869391502297155):2.1705015049195673):2.2207464570076425):1.0138266878874322,((Exocelina_ater_Australia_MB2669:2.406945805987742,Exocelina_simplex_Australia_MB1445:2.406945805987742):12.299543931659532,(Exocelina_sp_nov_9_australis_Australia_MB0296:12.880210440098747,((Exocelina_parvula_Hawaii_MB1261:9.291565144891857,(Exocelina_shizong_China_MB0050:8.654699235952542,((Exocelina_creuxorum_New_Caledonia_MB0406:2.0670508991930183,(Exocelina_feryi_New_Caledonia_MB0142:0.37394105201374156,Exocelina_leae_New_Caledonia_MB0037:0.37394105201374156):1.6931098471792776):5.516209409018208,((Exocelina_aubei_New_Caledonia_MB0693:2.0894957560396983,Exocelina_cheesmaniae_Vanuatu_MB1396:2.0894957560396983):4.886122523723703,(((Exocelina_brownei_New_Caledonia_MB0139:3.4391077951433076,(Exocelina_flammi_New_Caledonia_MB0170:0.5184575553523165,Exocelina_perfecta_New_Caledonia_MB0168:0.5184575553523165):2.9206502397909957):0.4867503557710009,(Exocelina_remyi_New_Caledonia_MB0132:3.2262474478564585,(Exocelina_nielsi_New_Caledonia_MB0166:2.654496504516621,(Exocelina_poallabauerae_New_Caledonia_MB0036:1.026741682991345,(Exocelina_gelima_New_Caledonia_MB0403:0.6516970038781151,(Exocelina_barbarae_New_Caledonia_MB0121:0.4149831085384818,Exocelina_bimaculata_New_Caledonia_MB0163:0.4149831085384818):0.23671389533966286):0.37504467911320305):1.6277548215252804):0.5717509433398442):0.6996107030578411):1.3068026242937671,((Exocelina_commatifera_New_Caledonia_MB0090:2.5401062910568184,(Exocelina_simoni_New_Caledonia_MB0020:1.8471030803910058,(Exocelina_burwelli_New_Caledonia_MB0409:1.362639486664613,(Exocelina_gaulorum_New_Caledonia_MB0123:0.32403749521180897,Exocelina_niklasi_New_Caledonia_MB0039:0.32403749521180897):1.0386019914528004):0.48446359372640035):0.6930032106658):1.5536060319629383,(Exocelina_brunoi_New_Caledonia_MB0019:2.895052114637281,(Exocelina_staneki_New_Caledonia_MB0413:2.651894333967331,(Exocelina_charlottae_New_Caledonia_MB0254:2.1128367096641214,(Exocelina_rotteri_New_Caledonia_MB0135:1.6415610103758358,(Exocelina_interrupta_New_Caledonia_MB0253:0.9002150517098456,Exocelina_subjecta_New_Caledonia_MB0130:0.9002150517098456):0.7413459586659898):0.4712756992883149):0.5390576243032124):0.2431577806699492):1.1986602083824578):1.1389484521883269):1.7429575045553367):0.6076420284478141):1.0714389277413376):0.6368659089392761):1.9622204151332223,((Exocelina_abditus_Australia_MB0297:7.207863220581607,Exocelina_elongatulus_Australia_MB1850:7.207863220581607):2.8352556755900977,(((Exocelina_kinibeli_New_Guinea_MB1379:0.894002324264641,Exocelina_ullrichi_New_Guinea_MB1253:0.894002324264641):3.72675245027053,(((Exocelina_menyamya_New_Guinea_MB1377:1.175937194237163,Exocelina_pusilla_New_Guinea_MB1364:1.175937194237163):2.5533103454108055,(Exocelina_mekilensis_New_Guinea_MB0686:3.3362974300529844,((Exocelina_cyclops_New_Guinea_MB3330:1.2624968169894977,(Exocelina_adalbert_New_Guinea_MB1297:0.6403687946155523,Exocelina_bewani_New_Guinea_MB1296:0.6403687946155523):0.6221280223739172):1.4723292092203741,(Exocelina_koroba_New_Guinea_MB1292:2.269642199809501,(Exocelina_ibalimi_New_Guinea_MB0657:1.7466066090006718,(Exocelina_piusi_New_Guinea_MB4921:1.20975275244725,(Exocelina_fume_New_Guinea_MB4169:0.3678968453302449,Exocelina_pseudokeki_New_Guinea_MB1530:0.3678968453302449):0.8418559071170058):0.5368538565534129):0.5230355908088336):0.4651838264003749):0.6014714038431097):0.39295010959501875):0.3500386803957012,((Exocelina_hagenensis_New_Guinea_MB1360:1.5206528094559637,(Exocelina_mendiensis_New_Guinea_MB1337:0.9287975871642652,(Exocelina_ambuaensis_New_Guinea_MB1290:0.604239510298642,(Exocelina_desii_New_Guinea_MB1399:0.30793872643835085,Exocelina_simbaijimi_New_Guinea_MB3312:0.30793872643835085):0.29630078386029324):0.32455807686564764):0.5918552222916669):1.823125305556729,((Exocelina_yoginofi_New_Guinea_MB1302:1.4678160289056856,(Exocelina_inengensis_New_Guinea_MB3309:0.1377213396091099,Exocelina_simbaiensis_New_Guinea_MB3315:0.1377213396091099):1.3300946892965617):0.3599921031789284,(Exocelina_cernyi_New_Guinea_MB0066:1.0679610192775526,(Exocelina_may_New_Guinea_MB0662:0.6317919700931941,(Exocelina_ketembang_New_Guinea_MB0680:0.44749081825796905,Exocelina_sagatai_New_Guinea_MB0671:0.44749081825796905):0.18430115183522505):0.4361690491843665):0.7598471128070514):1.5159699829280529):0.7355081050310197):0.5414685544914937):0.7515754934521421,((((Exocelina_huon_New_Guinea_MB1313:0.45944155758603034,Exocelina_sosanikai_New_Guinea_MB3840:0.45944155758603034):2.5686945972159076,(Exocelina_mianminensis_New_Guinea_MB0688:2.500724328725337,(Exocelina_kokoda_New_Guinea_MB3405:1.4885664954136861,(Exocelina_haiaensis_New_Guinea_MB0265:1.0966651626229784,Exocelina_larsoni_New_Guinea_MB1299:1.0966651626229784):0.39190133279071016):1.0121578333116161):0.5274118260766318):1.067422276602053,((Exocelina_kailaki_New_Guinea_MB3409:0.9593573706063201,Exocelina_pulchella_New_Guinea_MB3408:0.9593573706063201):2.4049957588546063,(Exocelina_aipomek_New_Guinea_MB3726:2.8437425146089947,(((Exocelina_andensis_New_Guinea_MB1366:0.9553884500890306,Exocelina_posmani_New_Guinea_MB3406:0.9553884500890306):1.20082006646042,(Exocelina_jaseminae_New_Guinea_MB1382:1.7515339569129893,(Exocelina_woitapensis_New_Guinea_MB3399:1.2950486701077881,(Exocelina_varirata_New_Guinea_MB3303:0.4234330262414558,Exocelina_wareaga_New_Guinea_MB3404:0.4234330262414558):0.8716156438663372):0.4564852868052016):0.40467455963646515):0.3132695239042107,((Exocelina_oh_New_Guinea_MB0261:1.8035266714471825,(Exocelina_waraseraensis_New_Guinea_MB0257:0.6693849816348708,(Exocelina_bacchusi_New_Guinea_MB1521:0.33541541342842635,Exocelina_herzogensis_New_Guinea_MB1383:0.33541541342842635):0.3339695682064439):1.1341416898123098):0.43082060995273186,((Exocelina_marawaka_New_Guinea_MB1361:1.1474323726148004,((Exocelina_hintelmanii_New_Guinea_MB1367:0.45182482799170703,(Exocelina_kurumulensis_New_Guinea_MB1528:0.15500122503213218,Exocelina_pseudobroschii_New_Guinea_MB1520:0.15500122503213218):0.2968236029595738):0.21754354478400256,(Exocelina_broschii_New_Guinea_MB1300:0.4152585976113857,((Exocelina_engaensis_New_Guinea_MB1525:0.16207271332090223,Exocelina_pseudomarinae_New_Guinea_M1287:0.16207271332090223):0.14485308455060486,(Exocelina_kairongensis_New_Guinea_MB3313:0.07183137607559367,Exocelina_marinae_New_Guinea_MB1291:0.07183137607559367):0.23509442179591275):0.10833279973990717):0.254109775164295):0.47806399983909):0.6971439234395997,((Exocelina_monae_New_Guinea_MB1381:0.42348059890826256,Exocelina_pseudomonae_New_Guinea_MB1375:0.42348059890826256):1.051375541704981,((Exocelina_injiensis_New_Guinea_MB1376:0.9177412265915889,Exocelina_kabwumensis_New_Guinea_MB1285:0.9177412265915889):0.18169176143584187,(Exocelina_miriae_New_Guinea_MB1374:0.8396471594005263,(Exocelina_garaina_New_Guinea_MB3876:0.6116042391181294,(Exocelina_danae_New_Guinea_MB0673:0.43608755752854034,(Exocelina_patepensis_New_Guinea_MB1315:0.33123935251843023,(Exocelina_damantiensis_New_Guinea_MB1519:0.11342179748628045,Exocelina_madangensis_New_Guinea_MB1527:0.11342179748628045):0.2178175550321495):0.10484820501008041):0.17551668158959005):0.22804292028239426):0.25978582862690236):0.3754231525858416):0.369720155441156):0.389770985345516):0.23513075905372505):0.37426447415535113):0.5206106148519356):0.7312053019430595):0.629742155148703,(Exocelina_bagus_New_Guinea_MB4915:4.211413287986718,(Exocelina_ransikiensis_New_Guinea_MB1269:3.8754208599227393,(Exocelina_skalei_New_Guinea_MB4427:3.598784166864817,((Exocelina_munaso_New_Guinea_MB0255:2.2509631556895804,(Exocelina_astrophallus_New_Guinea_MB1529:0.6656004973644174,Exocelina_atowaso_New_Guinea_MB0267:0.6656004973644174):1.5853626583251677):0.8613116939230085,(((Exocelina_kisli_New_Guinea_MB1373:0.6724764924920803,Exocelina_knoepchen_New_Guinea_MB1303:0.6724764924920803):1.438730251508189,((Exocelina_vovai_New_Guinea_MB1372:0.9936370951277079,((Exocelina_bismarckensis_New_Guinea_MB1306:0.4246340471136376,Exocelina_gorokaensis_New_Guinea_MB1307:0.4246340471136376):0.39228496065970675,(Exocelina_pseudoedeltraudae_New_Guinea_MB1288:0.5540461517114613,Exocelina_sandaunensis_New_Guinea_MB0681:0.5540461517114613):0.2628728560618806):0.17671808735436523):0.38217400357970677,(Exocelina_bismarckensis_New_Guinea_MB1369:0.977415008854449,(Exocelina_bundiensis_New_Guinea_MB1398:0.8165036573732354,(Exocelina_edeltraudae_New_Guinea_MB1341:0.7147186980777722,(Exocelina_jimiensis_New_Guinea_MB3311:0.573342828885267,Exocelina_tariensis_New_Guinea_MB1289:0.573342828885267):0.14137586919250633):0.10178495929545961):0.16091135148121638):0.3983960898529366):0.7353956452928865):0.618555111940482,(((Exocelina_angginensis_New_Guinea_MB1272:0.7762064604506779,Exocelina_arfakensis_New_Guinea_MB1268:0.7762064604506779):0.6589033790538965,((Exocelina_batanta_New_Guinea_MB1277:0.562546931911482,Exocelina_hendrichi_New_Guinea_MB1321:0.562546931911482):0.30336813601148105,(Exocelina_evelyncheesmanae_New_Guinea_MB1276:0.503598237744985,Exocelina_pseudopolita_New_Guinea_MB1275:0.503598237744985):0.3623168301780073):0.5691947715816116):0.9768830546288245,((Exocelina_irianensis_New_Guinea_MB4907:0.8997600547062514,Exocelina_wondiwoiensis_New_Guinea_MB0056:0.8997600547062514):1.270648574539402,(Exocelina_pseudoeme_New_Guinea_MB3759:1.876389544146999,(Exocelina_lembena_New_Guinea_MB4922:1.5920241471366934,((Exocelina_mantembu_New_Guinea_MB0060:1.0796267371219186,Exocelina_pseudosoppi_New_Guinea_MB4916:1.0796267371219186):0.2513272717114261,(Exocelina_brahmiensis_New_Guinea_MB1298:1.2432393147151293,((Exocelina_pinocchio_New_Guinea_MB3321:0.7333307003214254,Exocelina_wannangensis_New_Guinea_MB3761:0.7333307003214254):0.39728766583734787,((Exocelina_bewaniensis_New_Guinea_MB1295:0.8541551536116023,Exocelina_unipo_New_Guinea_MB4903:0.8541551536116023):0.1774473774389651,((Exocelina_pseudobifida_New_Guinea_MB0659:0.5885284959471484,Exocelina_utowaensis_New_Guinea_MB4905:0.5885284959471484):0.3226674727774439,(Exocelina_kakapupu_New_Guinea_MB4912:0.7034172672058806,(Exocelina_soppi_New_Guinea_MB4911:0.38100927398686874,Exocelina_weylandensis_New_Guinea_MB4908:0.38100927398686874):0.32240799321900987):0.20777870151871403):0.12040656232594649):0.09901583510820888):0.11262094855631943):0.08771469411821942):0.26107013830335046):0.28436539701030394):0.2940190850986568):0.24158426488774065):0.31776896180736314):0.3825129936718277):0.4865093172522048):0.2766366930579176):0.33599242806398033):0.5138872985659724):0.6470296814346224):4.670788628184392):1.2106666638533614):1.6264248800736603):1.82627929754854):0.6387409112250477):15.215764719452869):4.5886257968042266):9.534626979849158):10.133493152227565):15.078412547357374):3.604342092467391):24.264044052012423);

**Supplementary Information S6**. Geography coding used to conduct the BioGeoBEARS analyses

164 6 (A C G V I H)

Exocelina_abditus_Australia_MB0297 100000

Exocelina_adalbert_New_Guinea_MB1297 001000

Exocelina_aipomek_New_Guinea_MB3726 001000

Exocelina_ambuaensis_New_Guinea_MB1290 001000

Exocelina_andensis_New_Guinea_MB1366 001000

Exocelina_angginensis_New_Guinea_MB1272 001000

Exocelina_arfakensis_New_Guinea_MB1268 001000

Exocelina_astrophallus_New_Guinea_MB1529 001000

Exocelina_ater_Australia_MB2669 100000

Exocelina_atowaso_New_Guinea_MB0267 001000

Exocelina_aubei_New_Caledonia_MB0693 010000

Exocelina_australiae_Australia_MB1932 100000

Exocelina_bacchusi_New_Guinea_MB1521 001000

Exocelina_bagus_New_Guinea_MB4915 001000

Exocelina_barbarae_New_Caledonia_MB0121 010000

Exocelina_batanta_New_Guinea_MB1277 001000

Exocelina_bewani_New_Guinea_MB1296 001000

Exocelina_bewaniensis_New_Guinea_MB1295 001000

Exocelina_bimaculata_New_Caledonia_MB0163 010000

Exocelina_bismarckensis_New_Guinea_MB1306 001000

Exocelina_bismarckensis_New_Guinea_MB1369 001000

Exocelina_boulvardi_Australia_MB1818 100000

Exocelina_brahmiensis_New_Guinea_MB1298 001000

Exocelina_broschii_New_Guinea_MB1300 001000

Exocelina_brownei_New_Caledonia_MB0139 010000

Exocelina_brunoi_New_Caledonia_MB0019 010000

Exocelina_bundiensis_New_Guinea_MB1398 001000

Exocelina_burwelli_New_Caledonia_MB0409 010000

Exocelina_cernyi_New_Guinea_MB0066 001000

Exocelina_charlottae_New_Caledonia_MB0254 010000

Exocelina_cheesmaniae_Vanuatu_MB1396 000100

Exocelina_commatifera_New_Caledonia_MB0090 010000

Exocelina_creuxorum_New_Caledonia_MB0406 010000

Exocelina_cyclops_New_Guinea_MB3330 001000

Exocelina_damantiensis_New_Guinea_MB1519 001000

Exocelina_danae_New_Guinea_MB0673 001000

Exocelina_desii_New_Guinea_MB1399 001000

Exocelina_edeltraudae_New_Guinea_MB1341 001000

Exocelina_elongatulus_Australia_MB1850 100000

Exocelina_engaensis_New_Guinea_MB1525 001000

Exocelina_evelyncheesmanae_New_Guinea_MB1276 001000

Exocelina_ferrugineus_Australia_MB1501 100000

Exocelina_feryi_New_Caledonia_MB0142 010000

Exocelina_flammi_New_Caledonia_MB0170 010000

Exocelina_fume_New_Guinea_MB4169 001000

Exocelina_gapa_Australia_MB1813 100000

Exocelina_garaina_New_Guinea_MB3876 001000

Exocelina_gaulorum_New_Caledonia_MB0123 010000

Exocelina_gelima_New_Caledonia_MB0403 010000

Exocelina_gorokaensis_New_Guinea_MB1307 001000

Exocelina_hagenensis_New_Guinea_MB1360 001000

Exocelina_haiaensis_New_Guinea_MB0265 001000

Exocelina_hendrichi_New_Guinea_MB1321 001000

Exocelina_herzogensis_New_Guinea_MB1383 001000

Exocelina_hintelmanii_New_Guinea_MB1367 001000

Exocelina_huon_New_Guinea_MB1313 001000

Exocelina_ibalimi_New_Guinea_MB0657 001000

Exocelina_inengensis_New_Guinea_MB3309 001000

Exocelina_injiensis_New_Guinea_MB1376 001000

Exocelina_interrupta_New_Caledonia_MB0253 010000

Exocelina_irianensis_New_Guinea_MB4907 001000

Exocelina_jaseminae_New_Guinea_MB1382 001000

Exocelina_jimiensis_New_Guinea_MB3311 001000

Exocelina_kabwumensis_New_Guinea_MB1285 001000

Exocelina_kailaki_New_Guinea_MB3409 001000

Exocelina_kairongensis_New_Guinea_MB3313 001000

Exocelina_kakapupu_New_Guinea_MB4912 001000

Exocelina_ketembang_New_Guinea_MB0680 001000

Exocelina_kinibeli_New_Guinea_MB1379 001000

Exocelina_kisli_New_Guinea_MB1373 001000

Exocelina_knoepchen_New_Guinea_MB1303 001000

Exocelina_kokoda_New_Guinea_MB3405 001000

Exocelina_koroba_New_Guinea_MB1292 001000

Exocelina_kurumulensis_New_Guinea_MB1528 001000

Exocelina_larsoni_New_Guinea_MB1299 001000

Exocelina_leae_New_Caledonia_MB0037 010000

Exocelina_lembena_New_Guinea_MB4922 001000

Exocelina_maculata_New_Caledonia_MB0415 010000

Exocelina_madangensis_New_Guinea_MB1527 001000

Exocelina_mantembu_New_Guinea_MB0060 001000

Exocelina_marawaka_New_Guinea_MB1361 001000

Exocelina_marinae_New_Guinea_MB1291 001000

Exocelina_may_New_Guinea_MB0662 001000

Exocelina_mekilensis_New_Guinea_MB0686 001000

Exocelina_melanaria_Australia_MB1911 100000

Exocelina_mendiensis_New_Guinea_MB1337 001000

Exocelina_menyamya_New_Guinea_MB1377 001000

Exocelina_mianminensis_New_Guinea_MB0688 001000

Exocelina_miriae_New_Guinea_MB1374 001000

Exocelina_monae_New_Guinea_MB1381 001000

Exocelina_monteithi_New_Caledonia_MB0292 010000

Exocelina_munaso_New_Guinea_MB0255 001000

Exocelina_nielsi_New_Caledonia_MB0166 010000

Exocelina_niklasi_New_Caledonia_MB0039 010000

Exocelina_novaecaledoniae_New_Caledonia_MB0137 010000

Exocelina_oh_New_Guinea_MB0261 001000

Exocelina_ouin_New_Caledonia_MB0410 010000

Exocelina_parvula_Hawaii_MB1261 000001

Exocelina_patepensis_New_Guinea_MB1315 001000

Exocelina_perfecta_New_Caledonia_MB0168 010000

Exocelina_pinocchio_New_Guinea_MB3321 001000

Exocelina_piusi_New_Guinea_MB4921 001000

Exocelina_poallabauerae_New_Caledonia_MB0036 010000

Exocelina_posmani_New_Guinea_MB3406 001000

Exocelina_pseudobifida_New_Guinea_MB0659 001000

Exocelina_pseudobroschii_New_Guinea_MB1520 001000

Exocelina_pseudoedeltraudae_New_Guinea_MB1288 001000

Exocelina_pseudoeme_New_Guinea_MB3759 001000

Exocelina_pseudokeki_New_Guinea_MB1530 001000

Exocelina_pseudomarinae_New_Guinea_M1287 001000

Exocelina_pseudomonae_New_Guinea_MB1375 001000

Exocelina_pseudopolita_New_Guinea_MB1275 001000

Exocelina_pseudosoppi_New_Guinea_MB4916 001000

Exocelina_pulchella_New_Guinea_MB3408 001000

Exocelina_punctipennis_Australia_1804 100000

Exocelina_pusilla_New_Guinea_MB1364 001000

Exocelina_ransikiensis_New_Guinea_MB1269 001000

Exocelina_rasilis_Australia_MB0086 100000

Exocelina_remyi_New_Caledonia_MB0132 010000

Exocelina_rotteri_New_Caledonia_MB0135 010000

Exocelina_sagatai_New_Guinea_MB0671 001000

Exocelina_sandaunensis_New_Guinea_MB0681 001000

Exocelina_shizong_China_MB0050 000010

Exocelina_simbaiensis_New_Guinea_MB3315 001000

Exocelina_simbaijimi_New_Guinea_MB3312 001000

Exocelina_simoni_New_Caledonia_MB0020 010000

Exocelina_simplex_Australia_MB1445 100000

Exocelina_skalei_New_Guinea_MB4427 001000

Exocelina_soppi_New_Guinea_MB4911 001000

Exocelina_sosanikai_New_Guinea_MB3840 001000

Exocelina_sp_nov_10_Australia_MB2536 100000

Exocelina_sp_nov_11_Australia_MB1882 100000

Exocelina_sp_nov_12_Australia_MB1903 100000

Exocelina_sp_nov_13_glyptus_Australia_MB1788 100000

Exocelina_sp_nov_14_glyptus_Australia_MB1770 100000

Exocelina_sp_nov_15_glyptus_Australia_MB1881 100000

Exocelina_sp_nov_16_glyptus_Australia_MB1836 100000

Exocelina_sp_nov_17_Australia_MB1747 100000

Exocelina_sp_nov_18_Australia_MB1910 100000

Exocelina_sp_nov_1_Australia_MB295 100000

Exocelina_sp_nov_2_Australia_MB1503 100000

Exocelina_sp_nov_3_Australia_MB2521 100000

Exocelina_sp_nov_4_Australia_MB1759 100000

Exocelina_sp_nov_5_Australia_MB1710 100000

Exocelina_sp_nov_6_Australia_MB282 100000

Exocelina_sp_nov_7_Australia_MB1717 100000

Exocelina_sp_nov_8_Australia_MB107 100000

Exocelina_sp_nov_9_australis_Australia_MB0296 100000

Exocelina_staneki_New_Caledonia_MB0413 010000

Exocelina_stygobiont_sp_rasjadi_Australia 100000

Exocelina_subjecta_New_Caledonia_MB0130 010000

Exocelina_tariensis_New_Guinea_MB1289 001000

Exocelina_ullrichi_New_Guinea_MB1253 001000

Exocelina_unipo_New_Guinea_MB4903 001000

Exocelina_utowaensis_New_Guinea_MB4905 001000

Exocelina_varirata_New_Guinea_MB3303 001000

Exocelina_vovai_New_Guinea_MB1372 001000

Exocelina_wannangensis_New_Guinea_MB3761 001000

Exocelina_waraseraensis_New_Guinea_MB0257 001000

Exocelina_wareaga_New_Guinea_MB3404 001000

Exocelina_weylandensis_New_Guinea_MB4908 001000

Exocelina_woitapensis_New_Guinea_MB3399 001000

Exocelina_wondiwoiensis_New_Guinea_MB0056 001000

Exocelina_yoginofi_New_Guinea_MB1302 001000

Notes: A, Australia; C, New Caledonia; G, New Guinea; V, Vanuatu; I, China; H, Hawaii.

**Supplementary Information S7**. R code used to conduct the TreePar analyses

library (TreePar)

tree<-read.tree(“Chrono.tre”)

x<-getx(tree)

bd.shifts.optim(x, sampling=c(164/210,1,1,1,1,1), grid=0.1, start=0, end=15, yule = FALSE, ME = FALSE, all = FALSE, posdiv = FALSE)

**Supplementary Information S8**. R code used to conduct the BAMM analyses

library(BAMMtools)

tree <- read.tree("Chrono.tre")

edata <- getEventData(tree, eventdata = "event_data.txt", burnin=0.1)

mcmcout <- read.csv("mcmc_out.txt", header=T)

plot(mcmcout$logLik ~ mcmcout$generation)

burnstart <- floor(0.1 * nrow(mcmcout))

postburn <- mcmcout[burnstart:nrow(mcmcout), ]

library(coda)

effectiveSize(postburn$N_shifts)

effectiveSize(postburn$logLik)

shift_probs <- summary(edata)

shift_probs

prior.name<-"name_prior_probs.txt"

mcmc.name<-"name_mcmc_out.txt"

computeBayesFactors(mcmc.name, prior.name, burnin=0.1)

plot.bammdata(edata, lwd=2, legend=T)

priorshifts <- getBranchShiftPriors(tree, prior.name)

css <- credibleShiftSet(edata, priorshifts, set.limit = 0.95)

css$number.distinct

summary(css)

plot.credibleshiftset(css)

best <- getBestShiftConfiguration(edata, priorshifts)

plot.bammdata(best, lwd = 2)

addBAMMshifts(best, cex=2.5)

priorshifts <- getBranchShiftPriors(edata, prior.name)

bftree <- bayesFactorBranches(edata, priorshifts)

plot.phylo(bftree, cex=0.2)

edgemax <- which(bftree$edge.length == max(bftree$edge.length))

bftree$edge[edgemax ,2]

allrates <- getCladeRates(edata)

mean(allrates$lambda)

quantile(allrates$lambda, c(0.05, 0.95))

cladeXrates<-getCladeRates(edata, node=X)

mean(cladeXrates$lambda)

quantile(nondolphinrate$lambda, c(0.05, 0.95))

noncladeXrates<-getCladeRates(edata, node=X, nodetype=”exclude”)

mean(noncladeXrates$lambda)

quantile(nondolphinrate$lambda, c(0.05, 0.95))

plot.new()

st <- max(branching.times(tree))

plotRateThroughTime(edata, intervalCol="black", avgCol="limegreen", start.time=st, ylim=c(0,1), cex.axis=2)

plot.new()

st <- max(branching.times(tree))

plotRateThroughTime(edata, node=X, intervalCol="black", avgCol="limegreen", start.time=st, ylim=c(0,1), cex.axis=2)

plot.new()

par(mfrow=c(1,3))

st <- max(branching.times(tree))

plotRateThroughTime(edata, node=X, intervalCol="black", avgCol="limegreen", start.time=st, ylim=c(0,1), cex.axis=2)

plotRateThroughTime(edata, node=X2, intervalCol="black", avgCol="limegreen", start.time=st, ylim=c(0,1), cex.axis=2)

plotRateThroughTime(edata, node=X3, intervalCol="black", avgCol="limegreen", start.time=st, ylim=c(0,1), cex.axis=2)

**Supplementary Information S7**. R code used to conduct the MuSSE analyses

library(ape)

library(picante)

library(diversitree)

tree<-read.tree("Chrono.tre")

trait.full<-read.table("Exo.txt")

trait<-trait.full$V2

names(trait)<-trait.full$V1

trait<-trait[tree$tip.label]

names(trait)<-tree$tip.label

statecols<-c("1"="springgreen1","2"="gold","3"="deepskyblue","4"="firebrick1")

plot(tree,tip.color=statecols[trait], no.margin=T, cex=0.3)

p<-starting.point.musse(tree, 4, yule=FALSE)

lik<-make.musse(tree, trait, 4, sampling.f=c(30/40, 102/150, 28/40, 3/3), strict=TRUE)

argnames(lik)

lik.allequal<-constrain(lik,lambda2~lambda1,lambda3~lambda1,lambda4~lambda1,mu2~mu1,mu3~mu1,mu4~mu1,q21~q12,q23~q12,q24~q12,q13~q12,q31~q12,q32~q12,q34~q12,q14~q12,q41~q12,q42~q12,q43~q12)

argnames(lik.allequal)

fit.allequal<-find.mle(lik.allequal, p[argnames(lik.allequal)])

fit.allequal$par

lik.lambda.free<-constrain(lik,mu2~mu1,mu3~mu1,mu4~mu1,q21~q12,q23~q12,q24~q12,q13~q12,q31~q12,q32~q12,q34~q12,q14~q12,q41~q12,q42~q12,q43~q12)

argnames(lik.lambda.free)

fit.lambda.free<-find.mle(lik.lambda.free, p[argnames(lik.lambda.free)])

fit.lambda.free$par

lik.mu.free<-constrain(lik,lambda2~lambda1,lambda3~lambda1,lambda4~lambda1,q21~q12,q23~q12,q24~q12,q13~q12,q31~q12,q32~q12,q34~q12,q14~q12,q41~q12,q42~q12,q43~q12)

argnames(lik.mu.free)

fit.mu.free<-find.mle(lik.mu.free, p[argnames(lik.mu.free)])

fit.mu.free$par

lik.q.free<-constrain(lik,lambda2~lambda1,lambda3~lambda1,lambda4~lambda1,mu2~mu1,mu3~mu1,mu4~mu1)

argnames(lik.q.free)

fit.q.free<-find.mle(lik.q.free, p[argnames(lik.q.free)])

fit.q.free$par

lik.lambda.mu.free<-constrain(lik,q21~q12,q23~q12,q24~q12,q13~q12,q31~q12,q32~q12,q34~q12,q14~q12,q41~q12,q42~q12,q43~q12)

argnames(lik.lambda.mu.free)

fit.lambda.mu.free<-find.mle(lik.lambda.mu.free, p[argnames(lik.lambda.mu.free)])

fit.lambda.mu.free$par

lik.lambda.q.free<-constrain(lik,mu2~mu1,mu3~mu1,mu4~mu1)

argnames(lik.lambda.q.free)

fit.lambda.q.free<-find.mle(lik.lambda.q.free, p[argnames(lik.lambda.q.free)])

fit.lambda.q.free$par

lik.mu.q.free<-constrain(lik,lambda2~lambda1,lambda3~lambda1,lambda4~lambda1)

argnames(lik.mu.q.free)

fit.mu.q.free<-find.mle(lik.mu.q.free, p[argnames(lik.mu.q.free)])

fit.mu.q.free$par

lik.all.free<-constrain(lik)

argnames(lik.all.free)

fit.all.free<-find.mle(lik.all.free, p[argnames(lik.all.free)])

fit.all.free$par

lik.lambda1.free<-constrain(lik,lambda3~lambda2,lambda4~lambda2,mu2~mu1,mu3~mu1,mu4~mu1,q21~q12,q23~q12,q24~q12,q13~q12,q31~q12,q32~q12,q34~q12,q14~q12,q41~q12,q42~q12,q43~q12)

argnames(lik.lambda1.free)

fit.lambda1.free<-find.mle(lik.lambda1.free, p[argnames(lik.lambda1.free)])

fit.lambda1.free$par

lik.mu1.free<-constrain(lik,lambda2~lambda1,lambda3~lambda1,lambda4~lambda1,mu3~mu2,mu4~mu2,q21~q12,q23~q12,q24~q12,q13~q12,q31~q12,q32~q12,q34~q12,q14~q12,q41~q12,q42~q12,q43~q12)

argnames(lik.mu1.free)

fit.mu1.free<-find.mle(lik.mu1.free, p[argnames(lik.mu1.free)])

fit.mu1.free$par

lik.q1.free<-constrain(lik,lambda2~lambda1,lambda3~lambda1,lambda4~lambda1,mu2~mu1,mu3~mu1,mu4~mu1,q32~q23,q34~q23,q24~q23,q42~q23,q43~q23)

argnames(lik.q1.free)

fit.q1.free<-find.mle(lik.q1.free, p[argnames(lik.q1.free)])

fit.q1.free$par

lik.lambda1mu1.free<-constrain(lik,lambda3~lambda2,lambda4~lambda2,mu3~mu2,mu4~mu2,q21~q12,q23~q12,q24~q12,q13~q12,q31~q12,q32~q12,q34~q12,q14~q12,q41~q12,q42~q12,q43~q12)

argnames(lik.lambda1mu1.free)

fit.lambda1mu1.free<-find.mle(lik.lambda1mu1.free, p[argnames(lik.lambda1mu1.free)])

fit.lambda1mu1.free$par

lik.lambda1q1.free<-constrain(lik,lambda3~lambda2,lambda4~lambda2,mu2~mu1,mu3~mu1,mu4~mu1,q32~q23,q34~q23,q24~q23,q42~q23,q43~q23)

argnames(lik.lambda1q1.free)

fit.lambda1q1.free<-find.mle(lik.lambda1q1.free, p[argnames(lik.lambda1q1.free)])

fit.lambda1q1.free$par

lik.mu1q1.free<-constrain(lik,lambda2~lambda1,lambda3~lambda1,lambda4~lambda1,mu3~mu2,mu4~mu2,q32~q23,q34~q23,q24~q23,q42~q23,q43~q23)

argnames(lik.mu1q1.free)

fit.mu1q1.free<-find.mle(lik.mu1q1.free, p[argnames(lik.mu1q1.free)])

fit.mu1q1.free$par

lik.lambda1mu1q1.free<-constrain(lik,lambda3~lambda2,lambda4~lambda2,mu3~mu2,mu4~mu2,q32~q23,q34~q23,q24~q23,q42~q23,q43~q23)

argnames(lik.lambda1mu1q1.free)

fit.lambda1mu1q1.free<-find.mle(lik.lambda1mu1q1.free, p[argnames(lik.lambda1mu1q1.free)])

fit.lambda1mu1q1.free$par

lik.lambda2.free<-constrain(lik,lambda3~lambda1,lambda4~lambda1,mu2~mu1,mu3~mu1,mu4~mu1,q21~q12,q23~q12,q24~q12,q13~q12,q31~q12,q32~q12,q34~q12,q14~q12,q41~q12,q42~q12,q43~q12)

argnames(lik.lambda2.free)

fit.lambda2.free<-find.mle(lik.lambda2.free, p[argnames(lik.lambda2.free)])

fit.lambda2.free$par

lik.mu2.free<-constrain(lik,lambda2~lambda1,lambda3~lambda1,lambda4~lambda1,mu3~mu1,mu4~mu1,q21~q12,q23~q12,q24~q12,q13~q12,q31~q12,q32~q12,q34~q12,q14~q12,q41~q12,q42~q12,q43~q12)

argnames(lik.mu2.free)

fit.mu2.free<-find.mle(lik.mu2.free, p[argnames(lik.mu2.free)])

fit.mu2.free$par

lik.q2.free<-constrain(lik,lambda2~lambda1,lambda3~lambda1,lambda4~lambda1,mu2~mu1,mu3~mu1,mu4~mu1,q31~q13,q34~q13,q14~q13,q41~q13,q43~q13)

argnames(lik.q2.free)

fit.q2.free<-find.mle(lik.q2.free, p[argnames(lik.q2.free)])

fit.q2.free$par

lik.lambda2mu2.free<-constrain(lik,lambda3~lambda1,lambda4~lambda1,mu3~mu1,mu4~mu1,q21~q12,q23~q12,q24~q12,q13~q12,q31~q12,q32~q12,q34~q12,q14~q12,q41~q12,q42~q12,q43~q12)

argnames(lik.lambda2mu2.free)

fit.lambda2mu2.free<-find.mle(lik.lambda2mu2.free, p[argnames(lik.lambda2mu2.free)])

fit.lambda2mu2.free$par

lik.lambda2q2.free<-constrain(lik,lambda3~lambda1,lambda4~lambda1,mu2~mu1,mu3~mu1,mu4~mu1,q31~q13,q34~q13,q14~q13,q41~q13,q43~q13)

argnames(lik.lambda2q2.free)

fit.lambda2q2.free<-find.mle(lik.lambda2q2.free, p[argnames(lik.lambda2q2.free)])

fit.lambda2q2.free$par

lik.mu2q2.free<-constrain(lik,lambda2~lambda1,lambda3~lambda1,lambda4~lambda1,mu3~mu1,mu4~mu1,q31~q13,q34~q13,q14~q13,q41~q13,q43~q13)

argnames(lik.mu2q2.free)

fit.mu2q2.free<-find.mle(lik.mu2q2.free, p[argnames(lik.mu2q2.free)])

fit.mu2q2.free$par

lik.lambda2mu2q2.free<-constrain(lik,lambda3~lambda1,lambda4~lambda1,mu3~mu1,mu4~mu1,q31~q13,q34~q13,q14~q13,q41~q13,q43~q13)

argnames(lik.lambda2mu2q2.free)

fit.lambda2mu2q2.free<-find.mle(lik.lambda2mu2q2.free, p[argnames(lik.lambda2mu2q2.free)])

fit.lambda2mu2q2.free$par

lik.lambda3.free<-constrain(lik,lambda2~lambda1,lambda4~lambda1,mu2~mu1,mu3~mu1,mu4~mu1,q21~q12,q23~q12,q24~q12,q13~q12,q31~q12,q32~q12,q34~q12,q14~q12,q41~q12,q42~q12,q43~q12)

argnames(lik.lambda3.free)

fit.lambda3.free<-find.mle(lik.lambda3.free, p[argnames(lik.lambda3.free)])

fit.lambda3.free$par

lik.mu3.free<-constrain(lik,lambda2~lambda1,lambda3~lambda1,lambda4~lambda1,mu2~mu1,mu4~mu1,q21~q12,q23~q12,q24~q12,q13~q12,q31~q12,q32~q12,q34~q12,q14~q12,q41~q12,q42~q12,q43~q12)

argnames(lik.mu3.free)

fit.mu3.free<-find.mle(lik.mu3.free, p[argnames(lik.mu3.free)])

fit.mu3.free$par

lik.q3.free<-constrain(lik,lambda2~lambda1,lambda3~lambda1,lambda4~lambda1,mu2~mu1,mu3~mu1,mu4~mu1,q21~q12,q24~q12,q14~q12,q41~q12,q42~q12)

argnames(lik.q3.free)

fit.q3.free<-find.mle(lik.q3.free, p[argnames(lik.q3.free)])

fit.q3.free$par

lik.lambda3mu3.free<-constrain(lik,lambda2~lambda1,lambda4~lambda1,mu2~mu1,mu4~mu1,q21~q12,q23~q12,q24~q12,q13~q12,q31~q12,q32~q12,q34~q12,q14~q12,q41~q12,q42~q12,q43~q12)

argnames(lik.lambda3mu3.free)

fit.lambda3mu3.free<-find.mle(lik.lambda3mu3.free, p[argnames(lik.lambda3mu3.free)])

fit.lambda3mu3.free$par

lik.lambda3q3.free<-constrain(lik,lambda2~lambda1,lambda4~lambda1,mu2~mu1,mu3~mu1,mu4~mu1,q21~q12,q24~q12,q14~q12,q41~q12,q42~q12)

argnames(lik.lambda3q3.free)

fit.lambda3q3.free<-find.mle(lik.lambda3q3.free, p[argnames(lik.lambda3q3.free)])

fit.lambda3q3.free$par

lik.mu3q3.free<-constrain(lik,lambda2~lambda1,lambda3~lambda1,lambda4~lambda1,mu2~mu1,mu4~mu1,q21~q12,q24~q12,q14~q12,q41~q12,q42~q12)

argnames(lik.mu3q3.free)

fit.mu3q3.free<-find.mle(lik.mu3q3.free, p[argnames(lik.mu3q3.free)])

fit.mu3q3.free$par

lik.lambda3mu3q3.free<-constrain(lik,lambda2~lambda1,lambda4~lambda1,mu2~mu1,mu4~mu1,q21~q12,q24~q12,q14~q12,q41~q12,q42~q12)

argnames(lik.lambda3mu3q3.free)

fit.lambda3mu3q3.free<-find.mle(lik.lambda3mu3q3.free, p[argnames(lik.lambda3mu3q3.free)])

fit.lambda3mu3q3.free$par

lik.lambda4.free<-constrain(lik,lambda2~lambda1,lambda3~lambda1,mu2~mu1,mu3~mu1,mu4~mu1,q21~q12,q23~q12,q24~q12,q13~q12,q31~q12,q32~q12,q34~q12,q14~q12,q41~q12,q42~q12,q43~q12)

argnames(lik.lambda4.free)

fit.lambda4.free<-find.mle(lik.lambda4.free, p[argnames(lik.lambda4.free)])

fit.lambda4.free$par

lik.mu4.free<-constrain(lik,lambda2~lambda1,lambda3~lambda1,lambda4~lambda1,mu2~mu1,mu3~mu1,q21~q12,q23~q12,q24~q12,q13~q12,q31~q12,q32~q12,q34~q12,q14~q12,q41~q12,q42~q12,q43~q12)

argnames(lik.mu4.free)

fit.mu4.free<-find.mle(lik.mu4.free, p[argnames(lik.mu4.free)])

fit.mu4.free$par

lik.q4.free<-constrain(lik,lambda2~lambda1,lambda3~lambda1,lambda4~lambda1,mu2~mu1,mu3~mu1,mu4~mu1,q21~q12,q23~q12,q13~q12,q31~q12,q32~q12)

argnames(lik.q4.free)

fit.q4.free<-find.mle(lik.q4.free, p[argnames(lik.q4.free)])

fit.q4.free$par

lik.lambda4mu4.free<-constrain(lik,lambda2~lambda1,lambda3~lambda1,mu2~mu1,mu3~mu1,q21~q12,q23~q12,q24~q12,q13~q12,q31~q12,q32~q12,q34~q12,q14~q12,q41~q12,q42~q12,q43~q12)

argnames(lik.lambda4mu4.free)

fit.lambda4mu4.free<-find.mle(lik.lambda4mu4.free, p[argnames(lik.lambda4mu4.free)])

fit.lambda4mu4.free$par

lik.lambda4q4.free<-constrain(lik,lambda2~lambda1,lambda3~lambda1,mu2~mu1,mu3~mu1,mu4~mu1,q21~q12,q23~q12,q13~q12,q31~q12,q32~q12)

argnames(lik.lambda4q4.free)

fit.lambda4q4.free<-find.mle(lik.lambda4q4.free, p[argnames(lik.lambda4q4.free)])

fit.lambda4q4.free$par

lik.mu4q4.free<-constrain(lik,lambda2~lambda1,lambda3~lambda1,lambda4~lambda1,mu2~mu1,mu3~mu1,q21~q12,q23~q12,q13~q12,q31~q12,q32~q12)

argnames(lik.mu4q4.free)

fit.mu4q4.free<-find.mle(lik.mu4q4.free, p[argnames(lik.mu4q4.free)])

fit.mu4q4.free$par

lik.lambda4mu4q4.free<-constrain(lik,lambda2~lambda1,lambda3~lambda1,mu2~mu1,mu3~mu1,q21~q12,q23~q12,q13~q12,q31~q12,q32~q12)

argnames(lik.lambda4mu4q4.free)

fit.lambda4mu4q4.free<-find.mle(lik.lambda4mu4q4.free, p[argnames(lik.lambda4mu4q4.free)])

fit.lambda4mu4q4.free$par

lik.lambda.mu.q.constrained<-constrain(lik,lambda4~lambda1,lambda3~lambda2,mu4~mu1,mu3~mu2,q21~q12,q23~q12,q24~q12,q13~q12,q31~q12,q32~q12,q34~q12,q14~q12,q41~q12,q42~q12,q43~q12)

argnames(lik.lambda.mu.q.constrained)

fit.lambda.mu.q.constrained<-find.mle(lik.lambda.mu.q.constrained, p[argnames(lik.lambda.mu.q.constrained)])

fit.lambda.mu.q.constrained$par

lik.lambda.mu.constrained.q.free<-constrain(lik,lambda4~lambda1,lambda3~lambda2,mu4~mu1,mu3~mu2)

argnames(lik.lambda.mu.constrained.q.free)

fit.lambda.mu.constrained.q.free<-find.mle(lik.lambda.mu.constrained.q.free, p[argnames(lik.lambda.mu.constrained.q.free)])

fit.lambda.mu.constrained.q.free$par

resultsGeography<-anova(

fit.allequal,all.lambda.free=fit.lambda.free,all.mu.free=fit.mu.free,all.q.free=fit.q.free,all.lambda.mu.free=fit.lambda.mu.free,all.lambda.q.free=fit.lambda.q.free,all.mu.q.free=fit.mu.q.free,all.lambda.mu.q.free=fit.all.free,

lambda1.free=fit.lambda1.free,mu1.free=fit.mu1.free,q1.free=fit.q1.free,lambda1mu1.free=fit.lambda1mu1.free,lambda1q1.free=fit.lambda1q1.free,mu1q1.free=fit.mu1q1.free,lambda1mu1q1.free=fit.lambda1mu1q1.free,

lambda2.free=fit.lambda2.free,mu2.free=fit.mu2.free,q2.free=fit.q2.free,lambda2mu2.free=fit.lambda2mu2.free,lambda2q2.free=fit.lambda2q2.free,mu2q2.free=fit.mu2q2.free,lambda2mu2q2.free=fit.lambda2mu2q2.free,

lambda3.free=fit.lambda3.free,mu3.free=fit.mu3.free,q3.free=fit.q3.free,lambda3mu3.free=fit.lambda3mu3.free,lambda3q3.free=fit.lambda3q3.free,mu3q3.free=fit.mu3q3.free,lambda3mu3q3.free=fit.lambda3mu3q3.free,

lambda4.free=fit.lambda4.free,mu4.free=fit.mu4.free,q4.free=fit.q4.free,lambda4mu4.free=fit.lambda4mu4.free,lambda4q4.free=fit.lambda4q4.free,mu4q4.free=fit.mu4q4.free,lambda4mu4q4.free=fit.lambda4mu4q4.free)

resultsGeography

write.table(resultsGeography,file="MuSSE Constant-rate and Varying-rate results of Exocelina.csv",sep=" ",quote=FALSE,row.names=FALSE)
